# RNA binding by the glucocorticoid receptor attenuates dexamethasone-induced gene activation

**DOI:** 10.1038/s41598-023-35549-y

**Published:** 2023-06-09

**Authors:** Nickolaus C. Lammer, Humza M. Ashraf, Daniella A. Ugay, Sabrina L. Spencer, Mary A. Allen, Robert T. Batey, Deborah S. Wuttke

**Affiliations:** 1 Department of Biochemistry, University of Colorado, Boulder, CO 80309 USA; 2 BioFrontiers Institute, University of Colorado, Boulder, CO 80309 USA

**Keywords:** Computational biology and bioinformatics, Molecular biology

## Abstract

The glucocorticoid receptor (GR) is a ligand-activated transcription factor that regulates a suite of genes through direct binding of GR to specific DNA promoter elements. GR also interacts with RNA, but the function of this RNA-binding activity remains elusive. Current models speculate that RNA could repress the transcriptional activity of GR. To investigate the function of the GR-RNA interaction on GR’s transcriptional activity, we generated cells that stably express a mutant of GR with reduced RNA binding affinity and treated the cells with the GR agonist dexamethasone. Changes in the dexamethasone-driven transcriptome were quantified using 4-thiouridine labeling of RNAs followed by high-throughput sequencing. We find that while many genes are unaffected, GR-RNA binding is repressive for specific subsets of genes in both dexamethasone-dependent and independent contexts. Genes that are dexamethasone-dependent are activated directly by chromatin-bound GR, suggesting a competition-based repression mechanism in which increasing local concentrations of RNA may compete with DNA for binding to GR at sites of transcription. Unexpectedly, genes that are dexamethasone-independent instead display a localization to specific chromosomal regions, which points to changes in chromatin accessibility or architecture. These results show that RNA binding plays a fundamental role in regulating GR function and highlights potential functions for transcription factor-RNA interactions.

## Introduction

The glucocorticoid receptor (GR) is a hormone-activated transcription factor (TF) that regulates numerous genes associated with stress, apoptosis, and inflammation^1–5^. GR is often the target of treatments for inflammatory diseases like rheumatoid arthritis and chronic obstructive pulmonary disease and, more recently, in the treatment of severe COVID-19 cases^6–9^. Due to GR’s importance as a drug target, its activation and regulation have been extensively characterized. In the canonical model of GR activation, gene regulation by glucocorticoids starts in the cytoplasm, where GR binds to glucocorticoids that passively diffuse into the cytoplasm which triggers dissociation from chaperone proteins and translocation into the nucleus^3^. GR then binds to pseudo-palindromic glucocorticoid response elements (GREs) on chromatin as a dimer and recruits transcriptional machinery to initiate robust gene activation^3,10–16^.

**Figure 1:**
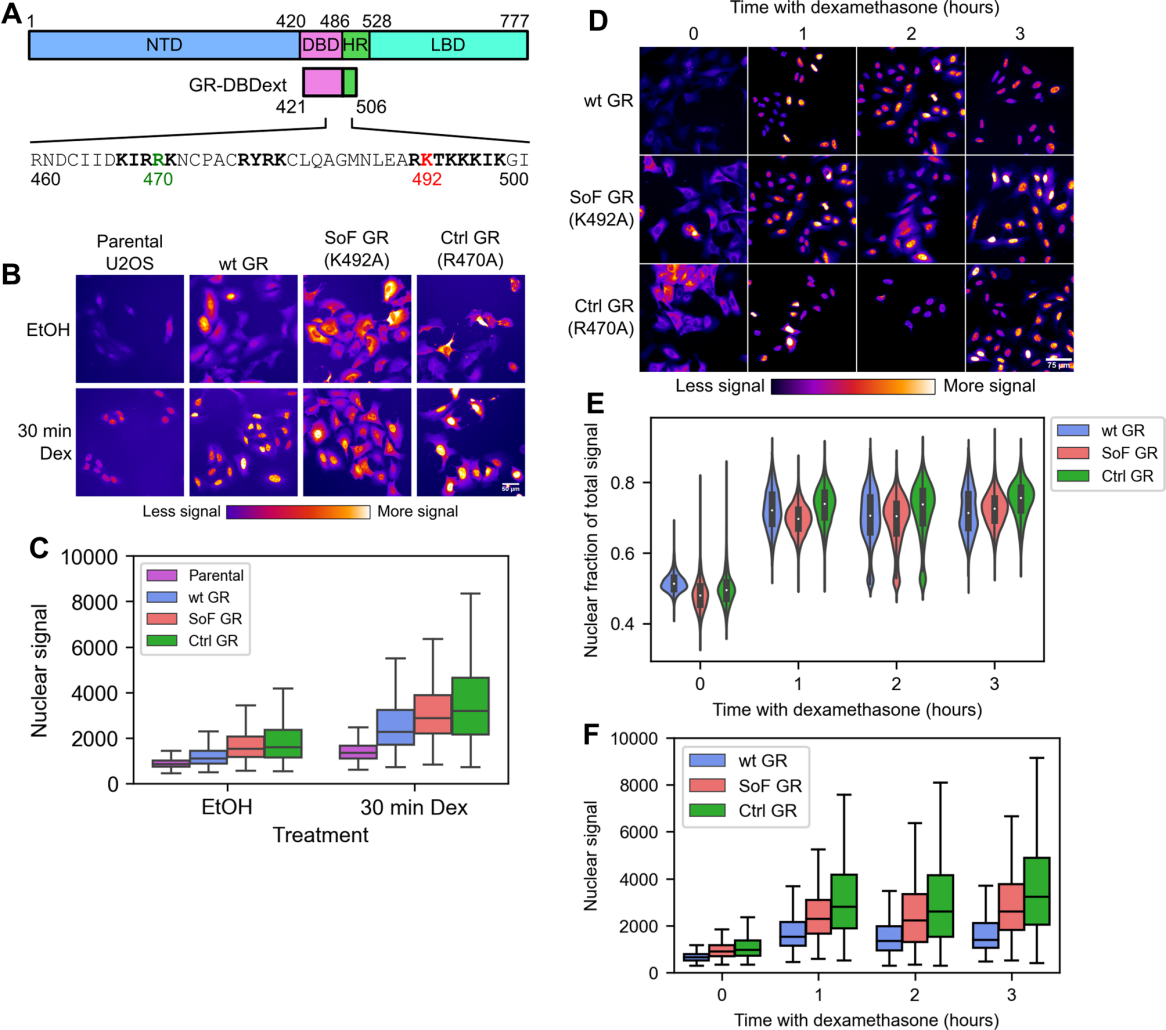
RNA binding does not regulate nuclear translocation of GR. (**A**) Domain map of full-length GR showing the N-terminal domain (NTD), DNA binding domain (DBD), hinge region (HR), and ligand binding domain (LBD). Below are the boundaries of the GR-DBDext construct and the sequence of amino acids 460–500 showing the tripartite nuclear localization signal (bold), SoF lysine residue (K492A, red), and Ctrl arginine residue (R470A, green)^11^. (**B**) Representative images of immunofluorescence using an anti-GR antibody with parental U2OS cells or FACS-sorted U2OS cell lines stably expressing wt GR-HaloTag, the SoF GR mutant, or Ctrl GR mutant selected for the transcriptomic assays. Cells were treated with 100 nM dexamethasone for 30 min or 0.01% ethanol (volume equivalent). Nuclear signal from each sample is quantified in (**C**). The intensity in all panels was scaled identically. (**C**) Box plot showing the nuclear fluorescent signal from immunofluorescence using an anti-GR antibody after 30 min of dexamethasone treatment. Outliers are hidden for clarity in comparing samples. (**D**) Representative images of immunofluorescence using an anti-GR antibody with U2OS cell lines stably expressing wt GR-HaloTag, the SoF GR mutant, or Ctrl GR mutant. Images are from a 3-h dexamethasone time course quantified in (**E**). The intensity in all panels was scaled identically. (**E**) Violin plots showing the fraction of total fluorescent signal that is nuclear from immunofluorescence using an anti-GR antibody. (**F**) Box plot showing the nuclear fluorescent signal from immunofluorescence using an anti-GR antibody after 3 h of dexamethasone treatment. Outliers are hidden for clarity in comparing samples.

Although many studies have focused on GR-DNA interactions that drive gene activation, there are several reports of GR-RNA interactions that exhibit repressive effects on GR and glucocorticoid-responsive genes. In one case, GR was found to initiate degradation of CCL2 and CCL7 mRNA by direct binding in the cytoplasm and subsequent recruitment of decay factors^17–19^. In another case, the non-coding RNA Gas5 directly repressed GR transcriptional activity when present in high amounts presumably by competing for interaction with GR’s DNA-binding domain (DBD)^2,20^. The broad array of GR-RNA binding targets and localization-specific functions motivated us to probe these interactions in vitro to clearly define the requirements of high-affinity binding^21^. From this study we determined that the GR-DBD containing additional residues from the C-terminal-adjacent hinge region (GR-DBDext, Fig. 1A) robustly binds hairpin RNAs with identical affinity as that exhibited to GRE DNA^21^. Binding between DNA and RNA ligands is fully competitive and, unlike DNA binding in which the most thermodynamically important interactions are in the N-terminal recognition helix, high-affinity RNA binding requires basic residues in the hinge region^21^.

Our in vitro results raise the tantalizing suggestion that any RNAs capable of adopting a hairpin structure could bind and regulate GR during glucocorticoid signaling by competing with DNA. However, no study to date has quantified the effect of RNA regulation on GR transcriptional activity by targeting GR’s RNA affinity directly. In this study, we assessed the function of GR-RNA binding in vivo by leveraging our in vitro results to generate a separation-of-function (SoF) mutant of GR with an 11-fold reduction in RNA affinity and 3.3-fold reduction in DNA affinity through mutation of K492 to alanine, a key basic residue in the hinge region^21^. We generated three U2OS cell lines stably expressing GR with a C-terminal HaloTag. The expressed GR-HaloTag is wild-type (wt) GR, the SoF GR mutant, or a control GR mutant (R470A) which has a moderate reduction in DNA affinity to match that of the SoF GR mutant^21,22^. We then measured changes in the dexamethasone-driven transcriptome associated with GR-RNA binding using 4-thiouridine (4sU) labeling of transcripts and high-throughput sequencing. Rather than finding a global impact on gene expression, we instead find that reducing GR-RNA affinity increases expression of two distinct subsets of genes. One set of genes is likely directly activated by GR based on co-occurrence with GR ChIP sites, which suggests RNA binding can play a repressive role at sites of transcriptional activation. The second set of genes is upregulated independent of dexamethasone treatment and shows localization to contiguous regions of chromatin, suggesting a role in modulating chromatin accessibility or architecture. These results present two distinct modes of GR regulation through binding RNA and provides insight into a novel element of GR’s complex regulatory pathways.

## Results

### Reducing the RNA binding affinity of GR does not impact GR nuclear translocation in response to dexamethasone

We previously identified a GR domain (GR-DBDext, residues 421–506, Fig. 1A) that exhibited tight and competitive binding to both a consensus GRE DNA and an RNA hairpin derived from the ncRNA Gas5^21^. This domain includes the classic DNA-binding domain with an 18 amino acid basic C-terminal extension into the adjacent hinge region (Fig. 1A). Previously, we performed an alanine scan of all basic residues within GR-DBDext and measured the binding affinities of these mutant proteins to consensus GRE DNA and the Gas5 RNA hairpin^21^. This comprehensive analysis revealed an RNA-binding separation-of-function (SoF) mutation at K492A (Fig. 1A). GR-DBDext with this mutation exhibits an 11-fold reduction in RNA affinity and a mere 3.3-fold reduction in DNA affinity^21^. As even this modest reduced DNA affinity could affect transcriptional activity, we used R470A as a control (Ctrl) mutant (Fig. 1A), which has a 4.8-fold reduction in DNA affinity but maintains wt RNA affinity.

To study the effects of reducing RNA binding affinity on GR function, we generated stable U2OS cell lines expressing the SoF GR mutant, Ctrl GR mutant, or wt GR with a C-terminal HaloTag^22^. GR constructs were integrated using the PiggyBac transposon system and puromycin was used for selection of successful genome integrations^23^. The U2OS cell line was chosen because several reports established very low expression of endogenous GR and an impaired response to dexamethasone in this cell line relative to glucocorticoid-sensitive cell lines, thus minimizing complications due to endogenous protein expression^16,24–26^. Because of this, a wide range of studies have used this cell line for investigating the impact of GR mutations on glucocorticoid signaling^14–16,25–27^. After transfection and selection, cells were sorted utilizing the HaloTag with conjugated dye through FACS and cell fractions were chosen to match nuclear GR abundance between cell lines after GR activation using immunofluorescence (Fig. 1B,C). Due to potential overlap of the SoF GR mutation and GR’s nuclear localization signal (Fig. 1A), we also confirmed the unhindered nuclear translocation (Fig. 1D,E) of all constructs during dexamethasone treatment^11^. Additionally, we observed that nuclear GR protein abundance is relatively constant for all GR constructs over 3 h of dexamethasone treatment (Fig. 1F). This observation is consistent with other studies that reported a decrease in GR abundance on time scales of 6–12 h of dexamethasone treatment, suggesting a lag time before appreciable degradation of GR begins^28,29^.

### GR binds RNA in vivo

**Figure 2:**
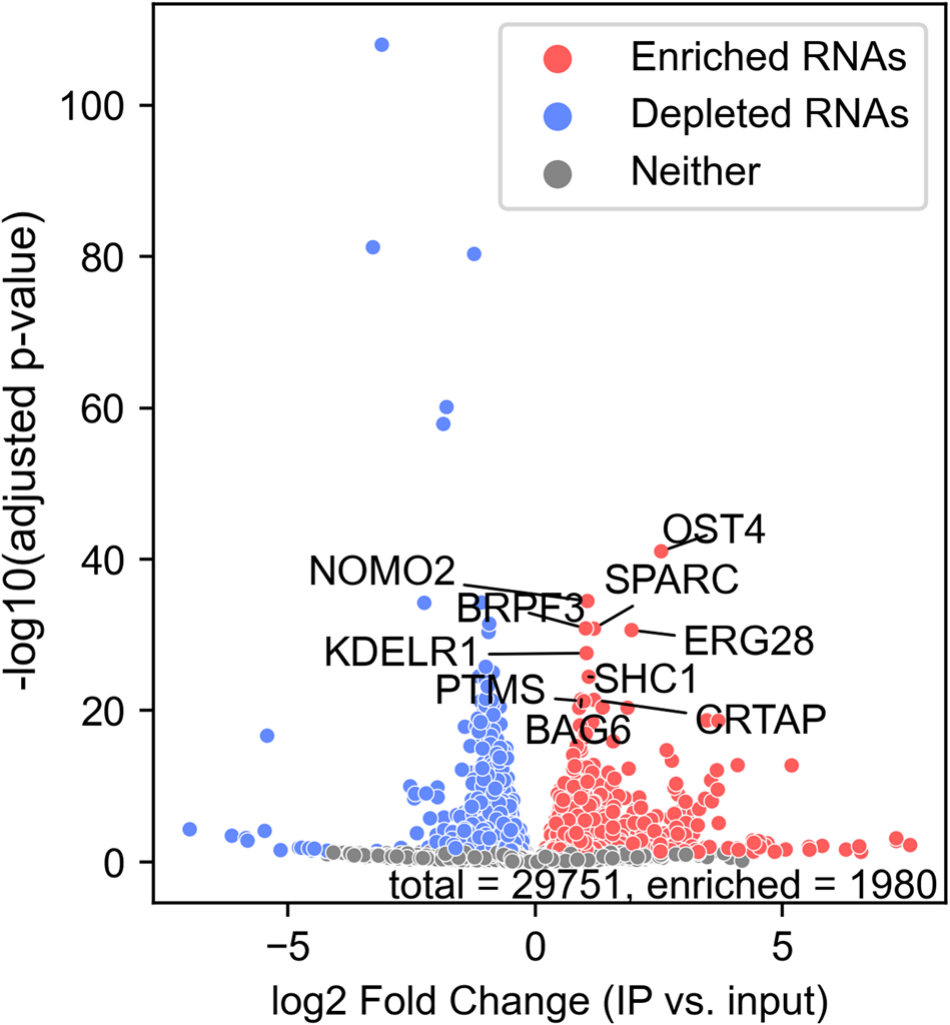
GR binds to RNAs in vivo. Volcano plot showing genes that are enriched or depleted in the GR RIP-seq. Cells were treated with 100 nM dexamethasone for 3 h. Log2 fold change is calculated as the RNA IP vs. input total RNA. Dot color indicates an adjusted p-value of fold change < 0.05 and a positive fold change (enriched, red) or negative fold change (depleted, blue). Labeled genes are the top 10 enriched genes ranked by adjusted p-value. The total number of genes in the plot and the number of enriched genes are listed. RIP-seq was performed in biological duplicate.

Before performing transcriptomic assays in SoF GR cells, we independently determined that wt GR binds robustly to RNAs in this cell-based system. RNA immunoprecipitation (RIP) with GR showing RNA binding has been performed previously, but not in the U2OS cell line^17^. To confirm that GR binds to RNAs in our cells, we performed RIP with UV crosslinking and sequencing. The covalent nature of both GR-RNA crosslinking and capture of GR-HaloTag allowed us to perform stringent denaturing washes that were designed to greatly reduce non-specific interactions^22,30^. The stringency of our HaloTag pulldown was confirmed using a silver stain and western blot against GR, where we found that the sample that remained on the resin after washing corresponded to the TEV cleavage product of GR-HaloTag (Fig. S1). After 3 h of dexamethasone treatment, we find that 1980 RNAs are enriched in our GR-HaloTag IP relative to the input total RNA (Fig. 2). The significance of the enriched genes in the context of GR biology is unclear, however the broad extent of GR-RNA binding supports our in vitro findings of structure-specificity to a common RNA secondary structure.

### RNA binding imparts distinct repression of two gene sets

Our initial hypothesis for the function of GR-RNA binding was that RNA functions as a competitive repressor of GR’s transcription factor activity by titrating GR off the DNA as RNA concentration increases, thus impacting the temporal response to glucocorticoid signaling. Our observations that DNA and RNA binding are competitive and that GR binds the common RNA hairpin motif without sequence specificity led to the prediction of a uniform but non-specific increase of transcriptional activity in the SoF GR cells as the ability of RNAs at sites of transcription to titrate GR off DNA is impaired^21^. To determine the effects of our SoF GR mutation on the dexamethasone-responsive transcriptome, we performed 4-thiouridine labeling of RNA upon continuous dexamethasone treatment followed by high-throughput sequencing (4sU-seq) of wt GR, SoF (K492A) GR, and Ctrl (R470A) GR-expressing cells. 4sU-seq was chosen to preferentially quantify transcripts expressed in response to dexamethasone^31,32^. The differences in GR’s transcriptional activity due to a reduction in RNA affinity were measured over a 3-h time course. PCA plots show that exon coverage for each sample varies by time in the first component (PC1) and by GR construct in the second component (PC2) (Fig. S2). These are the two factors we expected to be the most variable based on our experimental design, suggesting that the data does not vary due to unexpected factors.

We first examined how differential gene expression caused by dexamethasone treatment compared between the wt, SoF, and Ctrl GR cell lines. After three hours of dexamethasone treatment, 1413 genes are differentially expressed (adjusted p-value < 0.05) in all three cell lines (Fig. S3). Interestingly, each cell line also has a unique set of differentially expressed genes, which may indicate GR mutation-driven regulatory differences between cell lines. To probe how the GR mutations impact the expression of genes regulated by wt GR, we compared the abundance of 100 differentially expressed genes with the lowest adjusted p-values at 3 h of dexamethasone treatment in wt GR cells (Fig. S4). While these genes follow a similar pattern of differential expression between all GR constructs, some genes appear to have an increased magnitude of activation in the SoF GR cells after 3 h of dexamethasone treatment.

**Figure 3:**
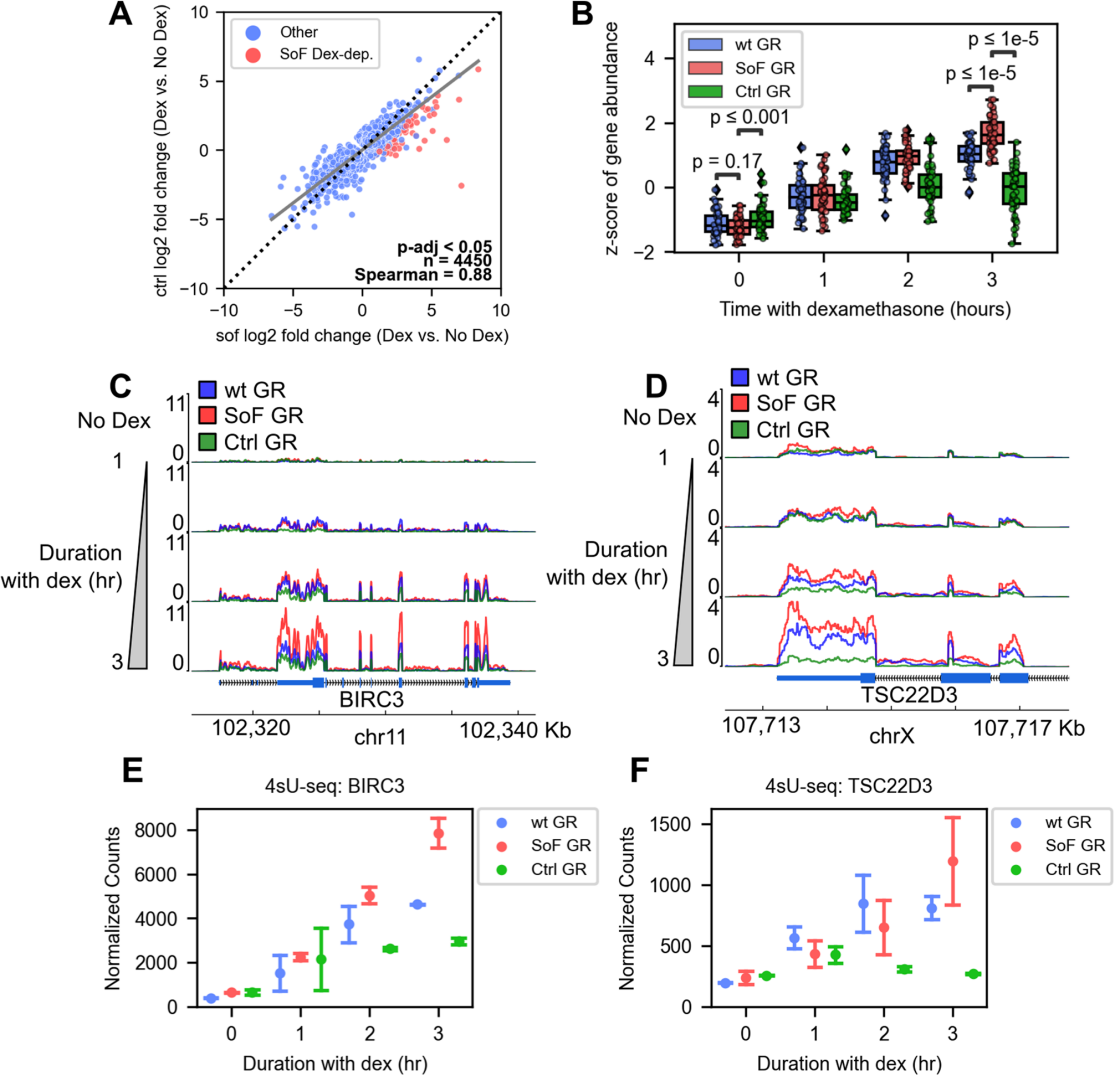
RNA binding is repressive for GR transcriptional activity at distinct subsets of genes. (**A**) Scatter plot showing the pairwise relation of gene fold changes between the SoF and Ctrl GR mutants at 3 h of 100 nM dexamethasone treatment. Dot color indicates statistical outliers picked as more activated with SoF GR (SoF Dex-dep. set). (**B**) Box and dot plot showing the z-score of gene abundance for SoF Dex-dep. genes over the 100 nM dexamethasone time course. Abundance is blue for the wt GR cells, red for the SoF GR mutant, and green for the Ctrl GR mutant. Significance testing was performed using Mann–Whitney tests. (**C**,**D**) Representative gene tracks for two SoF Dex-dep. genes: BIRC3 (**C**) and the 3’ end of TSC22D3 (**D**). Vertical progression corresponds to increasing 100 nM dexamethasone treatment time. Tracks display 4sU-seq reads normalized to transcripts per million plotted against chromosome coordinates. (**E**,**F**) Normalized 4sU-seq gene counts generated by DESeq2 for BIRC3 (**E**) and TSC22D3 (**F**) over the dexamethasone treatment time. Error bars in (**E**) and (**F**) represent 95% confidence intervals.

To further examine the effects of the SoF mutation on GR regulation, we plotted fold changes in differentially expressed genes at each time point pairwise between the SoF and Ctrl GR mutants and wt GR and visualized global differences in gene expression (Fig. S5). Using linear regression, we determined the largest discrepancy between SoF and Ctrl GR or wt GR regulation occurs at 3 h of dexamethasone treatment and so this time point was used for further comparisons (Fig. S5). The linear regression was fit using only genes that were statistically significant (adjusted p-value < 0.05) in a single sample or both within a sample pair. The higher gene fold-changes in SoF GR cells could be attributed to the absence of repressive RNA binding, similar to that reported for the GR-Gas5 ncRNA interaction^2,20^. The genes showing the largest difference in activation between SoF and Ctrl GR were isolated by finding statistical outliers based on log2 fold change and only including outliers below the regression line (Figs. 3A, S6). The regression line was used as a more stringent fold change cutoff as opposed to selecting any outlier genes with higher fold changes in SoF GR cells, which would also include genes with only slightly higher fold changes in SoF GR cells. Bivariate outliers were estimated using the minimum covariance determinant on genes whose fold changes have an adjusted p-value < 0.05 in a single sample or both within a sample pair. Doing this allowed us to identify a set of 50 dexamethasone-activated, high-confidence genes, which we term “SoF Dex-dep.,” that represent this repressive RNA effect after 3 h of dexamethasone treatment. Plotting the abundance of these genes over time for each GR construct illustrates the differences in transcriptional activation between SoF GR and both the Ctrl and wt GR (Fig. 3B). Representative genes from this set include BIRC3 (cIAP2), TSC22D3 (GILZ), CEBPB, and PDK4 (Figs. 3C–F, S7A–D) and the abundance over time of the 50 SoF Dex-dep. genes can be found in Fig. S8. The greater expression of BIRC3, TSC22D3, and PDK4 in the SoF GR cells was confirmed using RT-qPCR after 3 h of dexamethasone treatment (Fig. S7E–G). The activating effect of SoF GR and repressive effect of Ctrl GR relative to wt GR suggests that the effects of perturbations to RNA and DNA affinity are interdependent for this set of genes and that the level of de-repression due exclusively to loss of RNA binding may be even greater.

We also observed genes that were downregulated in SoF GR cells relative to Ctrl GR cells after 3 h of dexamethasone treatment (“SoF 3h Rep.”). These genes were chosen based on exhibiting a higher fold change in Ctrl GR cells (Fig. S9B). This gene set contains 23 genes and the difference in gene activation between SoF and Ctrl GR is less relative to the SoF Dex-dep. set (Fig. S9C). The abundance over time of these genes can be found in Fig. S10. The observation of dexamethasone-activated genes that are differentially up- or downregulated in SoF GR cells suggests that RNA binding does not serve a single, universal function in regulating GR’s transcriptional activity. Rather, a more complex gene family-specific process appears to be in place.

**Figure 4:**
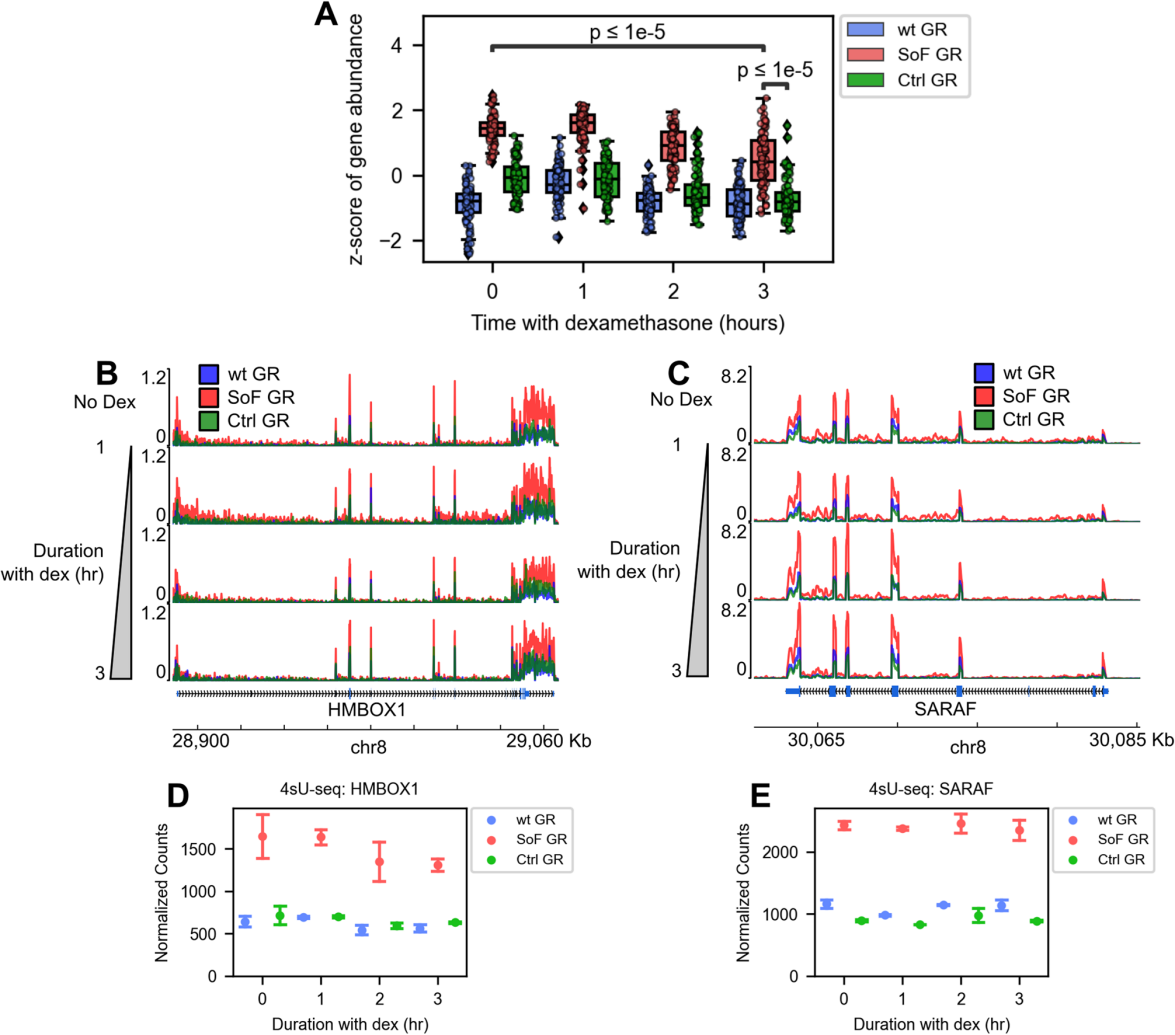
Reduction of GR-RNA affinity shows dexamethasone-independent activation at distinct subsets of genes. (**A**) Box and dot plot showing the z-score of gene abundance for genes activated in SoF GR cells independent of dexamethasone (SoF Dex-ind. set) over the 100 nM dexamethasone time course. Abundance is blue for the wt GR cells, red for the SoF GR mutant, and green for the Ctrl GR mutant. Significance testing was performed using Mann–Whitney tests. (**B**,**C**) Representative gene tracks for two SoF Dex-ind. genes: HMBOX1 (**B**) and SARAF (**C**). Vertical progression corresponds to increasing 100 nM dexamethasone treatment time. Tracks display 4sU-seq reads normalized to transcripts per million plotted against chromosome coordinates. (**D**,**E**) Normalized 4sU-seq gene counts generated by DESeq2 for HMBOX1 (**D**) and SARAF (**E**) over the dexamethasone treatment time. Error bars in (**D**) and (**E**) represent 95% confidence intervals.

In addition to the dexamethasone responsive genes, we also identified a several genes that are upregulated upon expression of SoF GR independent of dexamethasone treatment, defining a second distinct set of regulated genes characterized by this differential behavior. We grouped these genes and term them “SoF Dex-ind.” (Fig. 4A). Representative genes of this set include HMBOX1 and SARAF (Fig. 4B–E), along with DCTN6 and LINC01029 (Fig. S11A–D). The gene abundance heatmap for the 103 SoF Dex-ind. genes can be found in Fig. S12. The increased expression of HMBOX1, SARAF, and DCTN6 in the SoF GR cells compared to wt GR and Ctrl GR cells is supported by RT-qPCR with no dexamethasone (ethanol) and 3 h of dexamethasone treatment (Fig. S11E–G). Interestingly, analysis of the identity of these genes, as with the rest of the SoF Dex-ind. set, reveals no straightforward connection to known GR functions, suggesting a regulatory mechanism independent of GR’s transcriptional activity. Furthermore, we found genes that were constitutively downregulated in SoF GR cells compared to wt GR cells and term them “SoF Const. Rep.” (Fig. S9D). The abundance over time of these 95 genes is shown in Fig. S13. Like the SoF Dex-ind. genes, the nature of these genes is not clearly related to canonical GR functions. While this dexamethasone-independent regulation was unexpected, it is reminiscent of the glucocorticoid-independent gene regulation exhibited by the GRβ isoform^33,34^. This isoform was shown to regulate thousands of genes without glucocorticoid stimulation when expressed in U2OS cells and many of those genes do not overlap with GRα-regulated genes^34^. These results suggest, in contrast to a uniform repressive effect, that GR’s RNA-binding function is specific to subsets of genes in both dexamethasone-dependent and independent contexts.

### The RNA binding function of GR attenuates the dexamethasone-mediated activation of direct GR targets

**Figure 5:**
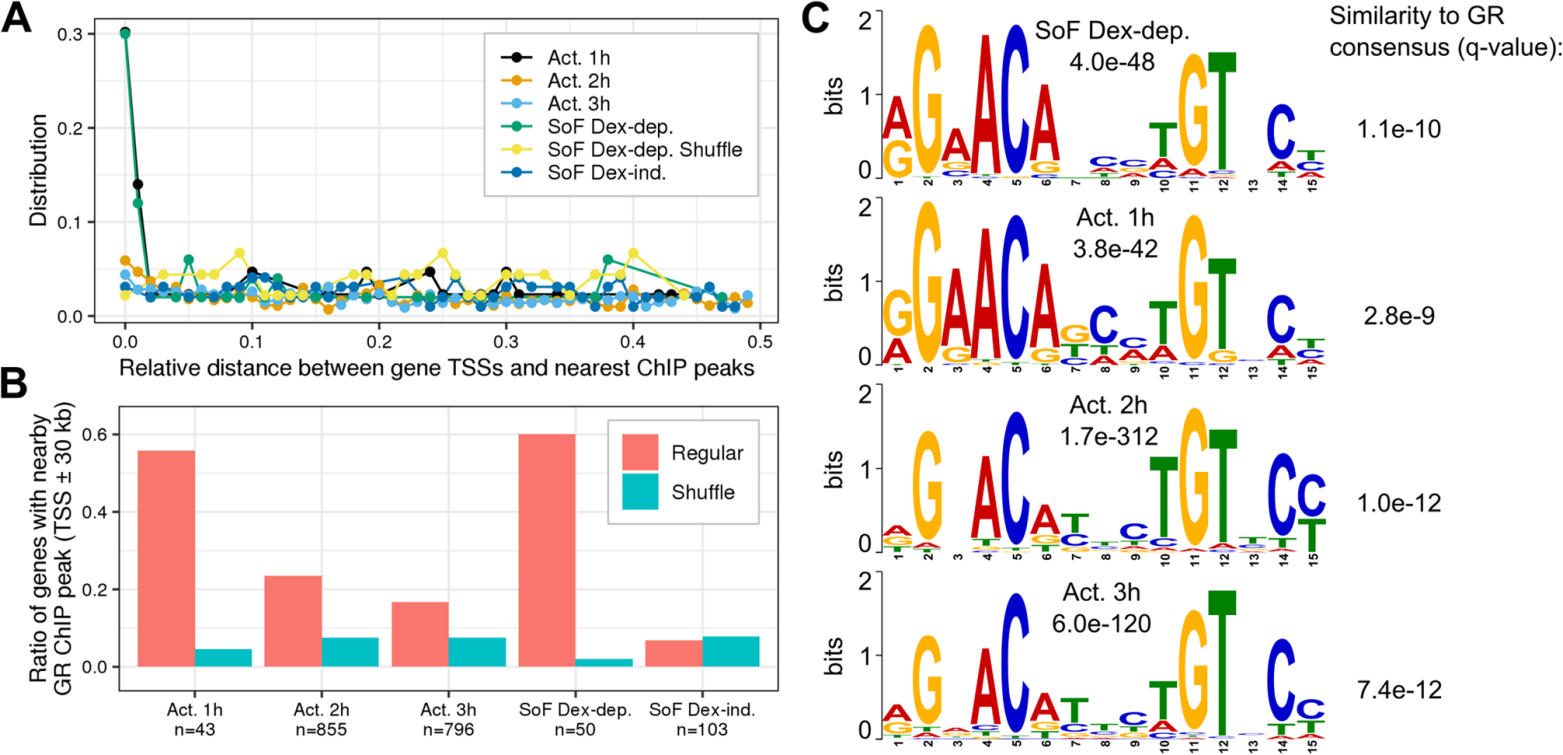
RNA binding represses direct GR targets. (**A**) Relative distance between ChIP peaks and gene TSSs in gene sets of interest. (**B**) Ratio of gene TSSs in each set with a GR ChIP peak within 30 kilobases with total number of genes in each set listed. The “Act.” sets refer to genes activated by 100 nM dexamethasone exclusively at 1, 2, or 3 h of treatment in wt GR cells. Shuffle refers to a version of those sets with the TSS regions randomized using bedtools shuffle, which serves as a negative control for GR ChIP co-occurrence^39^. (**C**) Motif logos from MEME motif discovery. Corresponding e-values from MEME are listed. Sequences were derived from GR ChIP peaks within 30 kb of TSSs. Similarity of each motif to the HOCOMOCO GR motif (GCR_HUMAN.H11MO.0.A) was quantified using Tomtom^42,89^.

The primary mechanism of direct transcriptional activation by GR is dimerization on chromatin at GREs^14,16,35,36^. One potential mechanism for RNA regulation of GR-driven gene activation would be for RNAs to compete with chromatin for binding to GR such that this mode of activation is repressed. To define the relationship between RNA binding and direct gene activation by GR, we probed the correlation of our SoF GR-upregulated gene sets with GR chromatin occupancy. To achieve this, we evaluated the co-occurrence of GR ChIP peaks from publicly available ChIP-seq datasets and the transcription start sites (TSSs) of genes from the SoF GR-upregulated gene sets. Co-occurrence of GR ChIP peaks and TSSs would suggest the genes in a given set are more likely directly activated by GR. By contrast, indirect gene activation, which could be initiated by the activation of other transcription factors, may show lower GR ChIP signal overlap and occur later in the dexamethasone time course relative to direct activation. To obtain ChIP peaks, we processed data from two previous studies that performed ChIP-seq in U2OS cells transfected with wt GR and used overlapping peaks present in both datasets (GEO accessions GSE109383 and GSE163398)^37^. Importantly, these ChIP-seq experiments were performed on timescales comparable to our 4sU-seq experiment. The TSSs were defined as the starting nucleotide of the gene body according to Gencode annotations to simplify the analysis^38^. To characterize the co-occurrence of ChIP signal and gene TSSs, we determined the relative distance between ChIP peaks and TSSs for our genes of interest as a smaller relative distance indicates more overlap between the two (Fig. 5A). To distinguish between direct and indirect gene activation, we also defined genes activated exclusively at each dexamethasone time point in wt GR cells (Act. 1–3 h). Specifically, these genes displayed a positive log2 fold-change when compared to the ethanol control sample with an adjusted p-value < 0.05. GR occupancy at activated genes decreases between 1 and 3 h of treatment, which suggests there is a shift from direct activation to predominantly indirect activation. Additionally, we included a shuffled set of gene TSSs to represent no co-occurrence with GR occupancy. Here, “shuffle” refers to the randomization of TSS genome positions using the bedtools shuffle function^39^. Thus, the shuffled sets serve as a visualization of non-association between TSSs and GR ChIP peaks as a negative control.

We find that SoF Dex-dep. genes overlap with GR binding sites to the same degree as genes activated at 1 h, suggesting that the SoF Dex-dep. gene set primarily contains direct GR targets. In contrast, the distribution for the SoF Dex-ind. set is similar to the randomized shuffle set, suggesting that GR does not directly regulate these genes via chromatin binding (Fig. 5A). To complement our distance distribution results, we plotted the ratio of genes in each set with a GR ChIP peak within 30 kb of the TSS. This analysis serves as a more intuitive quantification of the overlap between TSSs and GR occupancy. This window size was chosen because GR preferentially binds at enhancers distal to gene TSSs^1^. Doing this, we again observe that SoF Dex-dep. genes overlap with GR binding sites in a similar way to genes activated at 1 h and that the SoF Dex-ind. genes have no more overlap than the corresponding shuffled set (Fig. 5B). These results suggest that the SoF Dex-dep. genes are direct GR targets while the SoF Dex-ind. genes are activated through a mechanism independent of direct GR-chromatin binding.

The downregulation of SoF Dex-dep. genes when comparing Ctrl GR to wt GR (Fig. 3B) was expected given the direct nature of their activation by GR. However, the upregulation of these same genes when comparing SoF GR to wt GR was surprising. Since DNA and RNA binding to GR is competitive in vitro, these observations could suggest that the GREs at these genes are more sensitive to DNA affinity perturbations^21^. This would be most relevant for the Ctrl or wt GR where RNA can more effectively compete with DNA for binding to GR relative to SoF GR. A situation where this could arise is in the presence of a half-GRE motif, which binds to GR-DBDext with weaker affinity than the full GRE^16,21,40,41^. We addressed this by performing de novo motif discovery with sequences derived from GR ChIP peaks that overlap with TSSs from genes of interest within a 30 kb window. We find that the SoF Dex-dep. motifs are very similar to the consensus full GRE motif (Fig. 5C)^15,16,42^. This suggests that a weaker GRE motif may not be a mediator of gene attenuation through GR-RNA binding. We note that this analysis does not address the potential impact of nucleotides flanking these GREs or the presence of composite, non-consensus GREs, both of which could affect GR affinity to these sites relative to a consensus GRE^14,43–45^.

### Genes repressed by GR-RNA binding cluster on chromosomes 3, 7, and 8

**Figure 6:**
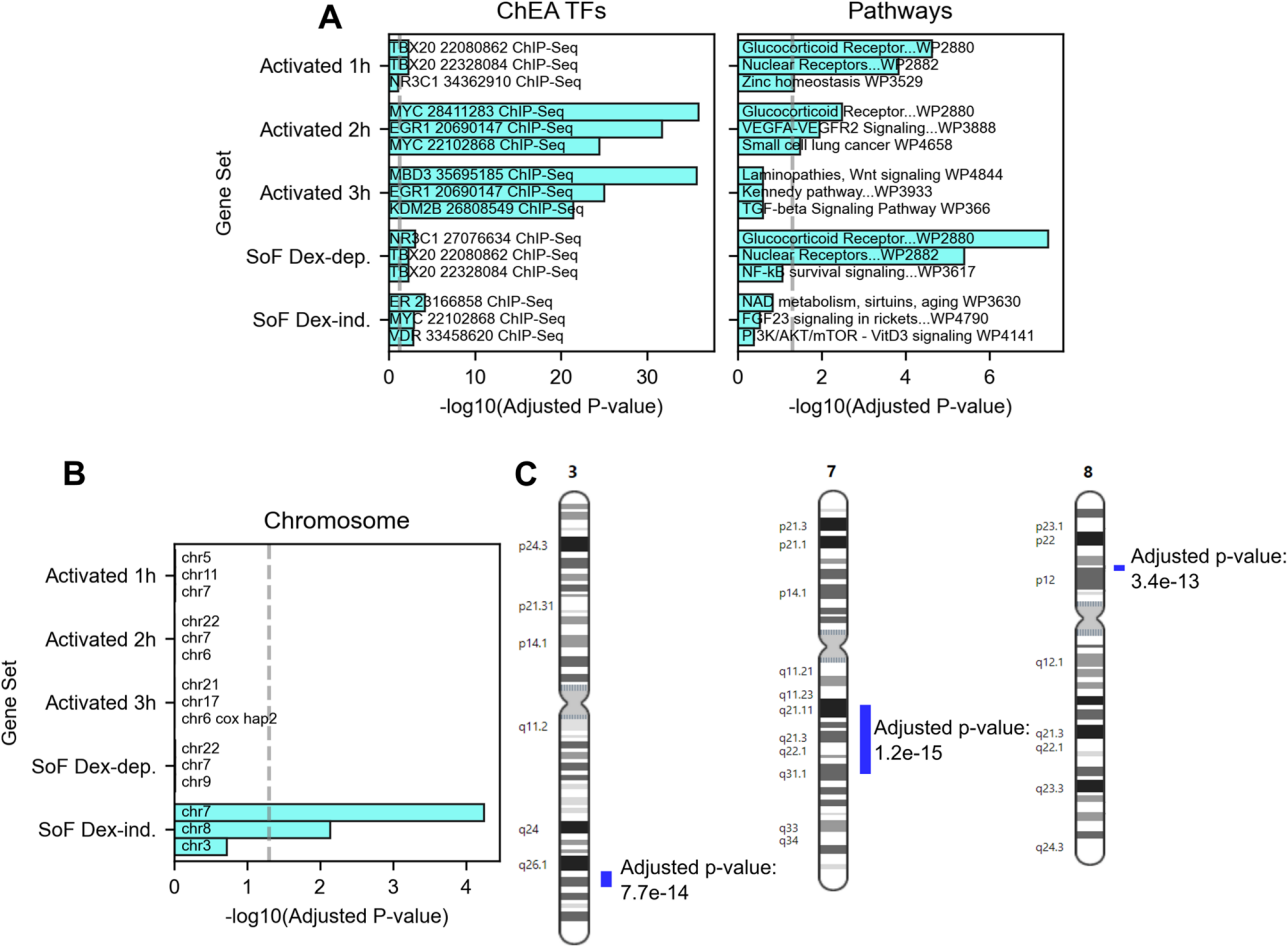
Genes activated in the absence of GR-RNA binding enrich for GR and NR pathways or specific chromosomes. (**A**,**B**) Enrichr gene set analysis with sets of interest for ChEA TF targets, WikiPathways (**A**), and chromosome (**B**)^46^. Plotted are the top three terms in each category based on the adjusted p-value. The pathway terms are shortened to fit in the plot. “Activated” refers to genes uniquely activated at 1, 2, or 3 h of 100 nM dexamethasone treatment. The dashed gray line indicates an adjusted p-value = 0.05. (**C**) Regions of chromosomal enrichment of SoF Dex-ind. genes based on output from the Positional Gene Enrichment tool^49^. Chromosomes 3, 7, and 8 are shown. Blue bars indicate enriched regions and their associated FDR-adjusted p-values are shown.

Our GR ChIP analysis strongly suggests that the SoF Dex-dep. genes are direct GR targets. To support this finding, we sought to determine the functional relationship between these genes and GR. We utilized Enrichr’s comprehensive gene set analysis to investigate if they are associated with other TFs or fall into specific pathways^46^. We first looked at SoF Dex-dep. genes and, as expected, they are enriched for both GR ChIP targets and glucocorticoid/nuclear receptor pathways (Fig. 6A). This supports our conclusion that RNA binding by GR reduces the transcriptional activation of direct and canonical GR targets. We also performed Enrichr analysis with the SoF 3h Rep. (repressed at 3 h, Fig. S10) and SoF Const. Rep. (constitutively repressed, Fig. S13) gene sets and only find ETS1 and POU2F1 ChIP targets to be significant, respectively (Fig. S14). Interestingly, POU2F1 (Oct-1) is known to interact with GR and contribute to gene activation at GREs with nearby octamer motifs^47,48^.

The SoF Dex-ind. genes do not correlate with GR ChIP signal, indicating that they are not direct GR targets, consistent with the fact that they are not responsive to dexamethasone. We next sought to determine common characteristics among SoF Dex-ind. genes that might point to their mechanism of action. Analysis of this gene set shows enrichment of non-GR TF targets with little functional correlation (Fig. 6A). While these genes do not display a canonical relationship to GR, the SoF Dex-ind. genes instead show a surprising positional correlation. About one-third of these genes are located contiguously on chromosomes 3, 7, and 8, whereas the other sets show no location enrichment (Fig. 6B). To support this finding, we used the Positional Gene Enrichment tool, which resulted in enrichment of the same chromosomes with defined enriched regions (Fig. 6C)^49^. This positional enrichment is unexpected and suggests a potential change in chromatin accessibility or architecture in the absence of GR-RNA binding independent of dexamethasone treatment.

## Discussion

Several studies have implicated RNA binding by GR in regulating expression of specific glucocorticoid-regulated genes, although the extent and significance of this activity is unknown^2,17–20^. Here, we specifically reduced GR-RNA affinity in cells and measured the impact on the dexamethasone-driven transcriptome. We find that distinct subsets of genes are further upregulated upon reduction of GR-RNA binding, both in response to and independent of dexamethasone treatment. Based on the trajectory of gene abundance, activation of dexamethasone-driven genes appears to be approaching its maximum at 3 h and that maximum level is higher upon attenuation of GR-RNA binding (Fig. S15). Consistent with this, a study of the dexamethasone-driven transcriptome in A549 cells shows that maximum gene activation was observed after about 3 h for some of these same genes^1^.

**Figure 7:**
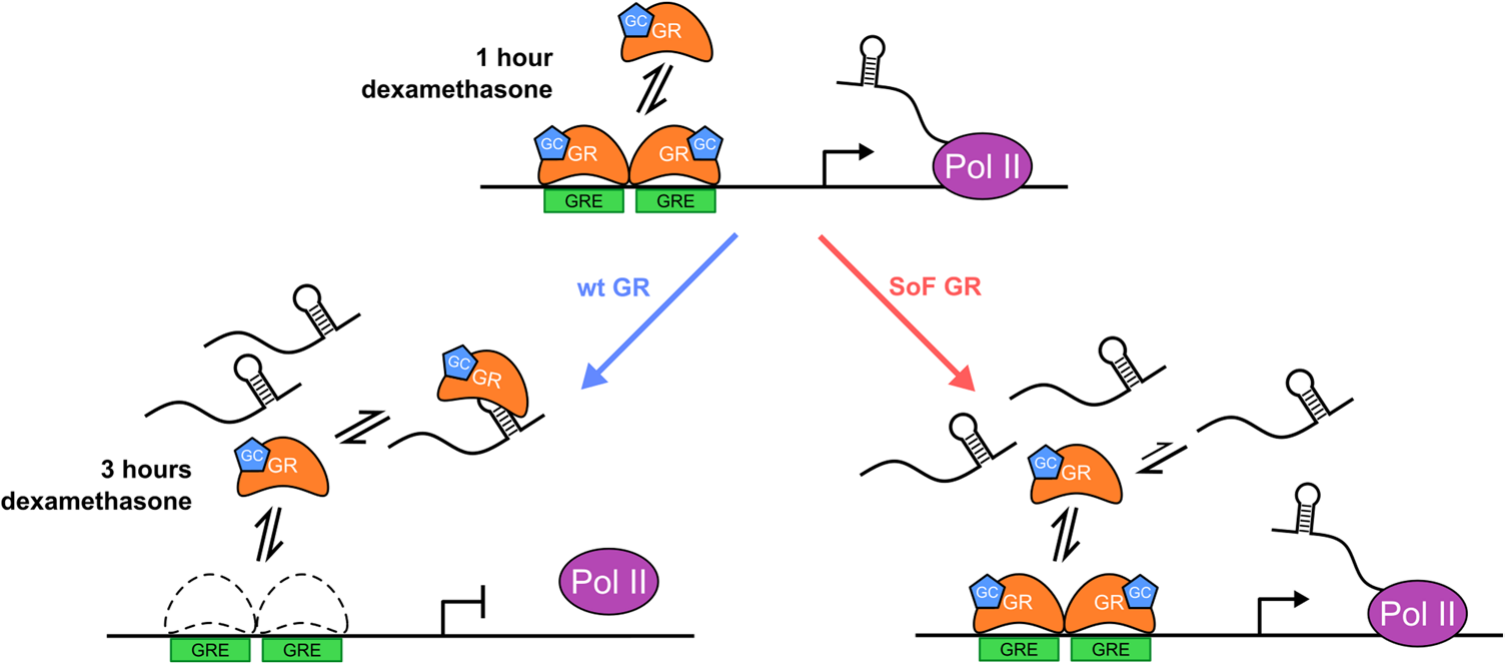
RNA limits activation of GR-bound genes. Model for repression of GR activity via RNA binding at sites of transcription. One hour after addition of dexamethasone, GR is present on chromatin at directly activated targets and transcription begins. After three hours of treatment, enough RNA is present to effectively compete GR off of chromatin and transcription reaches a maximum. However, if competitive binding between GR-DNA and GR-RNA complexes is skewed towards DNA binding, as in the case of the SoF GR, then the maximum for transcriptional activation will increase.

During continuous dexamethasone treatment, the maximum transcriptional response is dictated by the interplay between the residence time of GR on chromatin, proteasomal degradation of GR, and the binding of GR to chaperones^28,50–54^. Specifically, the proteasome is known to attenuate GR activation^28,50,51^. Additionally, perturbation of the proteasome or chaperone proteins affects the residence time of GR on chromatin, which is coupled to transcriptional output^52^. Given our results, we propose that RNA binding, either to the induced transcript or other proximal RNAs, could also similarly modulate GR’s residence time by competing with chromatin and repressing GR activity over continuous hormone stimulation (Fig. 7).

This activating effect of SoF GR was not universal, but instead affects just a subset of genes. While the origin of this specificity is unknown, based on our in vitro binding data and other in vivo studies, it may be that transcripts from this subset have a higher propensity for forming hairpin secondary structures that increases their affinity for GR binding^2,17,21^. We note, however, that we did not observe specific enrichment of these SoF Dex-dep. genes in our GR-HaloTag RIP dataset (Fig. S16). This could be due to the potentially transient nature of these interactions or that the pulldown captures the bulk GR population, when only a subset is engaged in transcriptional activity. Additionally, we did not specifically test for RNA binding deficiencies in vivo in this study, however, the data from our in vitro study strongly suggests that the SoF GR mutation reduces GR-RNA binding^21^. As a result, validation of the detailed mechanism of this regulation will require further investigation.

Many factors influence the interaction between GR and chromatin and subsequent gene regulation. Our study does not account for non-uniformity in DNA affinity changes between our mutant GR constructs and the varied GRE sequence space among GR-regulated genes, especially in conjunction with co-regulatory factors^3,14,15,21^. These affinities were characterized with a single consensus GRE and both the SoF and Ctrl GR constructs may bind differently between GRE sequences. As DNA binding is known to have allosteric effects on GR, binding at varied GREs could impact also the ability for RNA to compete for binding^14,15,55^.

Our data are consistent with and extend the effects of GR-RNA association noted in other studies of a limited set of RNAs^2,20^. The ncRNA Gas5 was found to likewise repress GR transcriptional activity by binding to its DBD. The magnitude of repression was directly related to the increased abundance of Gas5 during serum starvation^2,20^. Our results corroborate this repressive RNA effect with a GR-centric approach and extend these findings to the whole transcriptome under normal tissue culture conditions. Specifically, Kino et al. found that the SoF Dex-dep. genes BIRC3 (cIAP2) and TSC22D3 (GILZ) were also repressed by Gas5^2^. Other genes found to be repressed by Gas5 were not contained in our SoF Dex-dep. set, but this could be due to our stringent threshold for inclusion in this set or cell type-specific gene regulation. In another study, the same GR-K492A mutation we identified as our SoF GR mutation showed increased activation of a luciferase reporter with a murine mammary tumor virus (MMTV) promoter, a model steroid receptor transcriptional activation reporter^56,57^. These observations corroborate our more comprehensive conclusion that RNA binding by GR represses direct GR targets.

Notably, GR-RNA binding repressed expression of a second group of genes independent of dexamethasone treatment (SoF Dex-ind.) that are spatially contiguous on chromatin. While an interpretation of this observation is necessarily speculative, it suggests that GR-RNA binding could contribute to the maintenance of chromatin accessibility or architecture in the regions enriched for SoF Dex-ind. genes. While GR is thought to be mostly cytoplasmic in the absence of glucocorticoids, several studies have observed GR nuclear localization to occur without ligand and in coordination with cell cycle phases^3,10,11,58–62^. Indeed, in the absence of dexamethasone, about half of the total anti-GR fluorescent signal in our immunofluorescence experiment was nuclear (Fig. 1E). Additionally, there is recent precedent for the role of RNA in maintaining nuclear compartments^63,64^. These results suggest that nuclear GR contributes to the maintenance of chromatin organization through association with RNA.

The region of GR critical for RNA binding overlaps with the nuclear localization signal and is prone is post-translational modification (Fig. 1A). Specifically, the CLOCK/BMAL1 circadian rhythm-regulated heterodimer was found to acetylate the lysines K480, K492, K494, and K495, though a direct connection between acetylation and nuclear localization has not been established^11,56^. Mutation of K492 to alanine, as in our SoF GR construct, would remove a potential acetylation site and could modulate GR activity separately from RNA binding. However, recent studies have suggested that acetylation of lysines in the RNA-binding proteins G3BP1, Tau, and FUS reduces their affinities to RNA^65–67^. These findings raise the intriguing possibility that RNA binding by GR could be regulated by modification of the basic residues critical for RNA-binding activity. The resulting reduced RNA affinity may mimic our results with SoF GR and potentiate binding to chromatin.

Competitive DNA/RNA binding likely extends to other nuclear receptors and other families of transcription factors as well. The region of basic residues implicated in GR-RNA binding is highly conserved among other nuclear receptors (Fig. S17) and there is a growing body of evidence for the regulation of estrogen receptor α (ERα) via RNA binding^68–70^. Recently it was found that ERα, like GR, binds RNA hairpins with high affinity and RNA also competes for binding with DNA^68^. Ablation of ERα-RNA affinity could have the same effect on the estradiol-driven transcriptome that we found with GR. However, the only established relationship thus far between ERα-RNA binding and the estradiol-driven transcriptome is recruitment to sites of repression via eRNA^69^. It is also becoming increasingly evident that this RNA binding phenomenon extends to many other TFs. YY1, SMAD3, SOX2, and TFIIIA among others have been shown to interact with RNA with the potential to modulate their transcriptional function, although not all of these TFs bind DNA and RNA competitively^71–77^. RNA-bound proteome studies have identified many more TFs, including some that contain arginine- and lysine-rich motifs akin to the basic RNA-binding region in GR that associated directly with RNA^78–80^. Based on our results with GR, negative feedback caused by RNA binding may be a fundamental feature of TFs to regulate transcriptional activity.

## Methods

### Cell culture and generation of stable GR-HaloTag-expressing cell lines

Parental U2OS cells were a gift from Dr. Amy Palmer’s lab (CU-Boulder), originally from ATCC (HTB-96). Cells were cultured in DMEM supplemented with 10% fetal bovine serum (FBS, Peak Serum Inc.) and 1 X penicillin–streptomycin (100 U/ml, 100 µg/ml, Life Technologies Corporation) in a humidified incubator maintained at 37 °C and 5% CO_2_. For experiments with dexamethasone treatment, charcoal-stripped FBS was used to remove background hormones. For every 50 mL FBS, 1 g activated charcoal (MilliporeSigma) was washed with sterile 40 mL 1 X PBS. PBS was aspirated and then 37.5 mg of dextran 70 (Tokyo Chemical Industries) and 50 mL FBS was added. FBS was rocked for 1 h at 4 °C and sterile filtered.

To generate stable cell lines, we first cloned wild-type or mutant (K492A or R470A) GR C-terminally tagged with HaloTag into the BamHI and EcoRI sites on the PiggyBac plasmid PB-CMV-MCS-EF1α-Puro (System Biosciences, PB510B-1)^22,23^. Cell lines were generated using the PiggyBac Transposon system via transfection with TransIT-LT1 transfection reagent (Mirus Bio). After 2 μg/mL puromycin selection, cells were sorted using FACS with HaloTag-conjugated Janelia Fluor 646 as the fluorescent marker (Promega). Cells were sorted into low, low-medium, medium–high, and high intensity fractions based on the signal distribution. Cell fractions for transcriptomics assays were selected to match nuclear signal between GR constructs after 30-min dexamethasone treatment using immunofluorescence (performed as stated in the following section).

### Measurement of GR translocation with immunofluorescence

Prior to final plating, each cell line was grown on a 6 cm dish using DMEM supplemented with 10% charcoal-stripped FBS and 1 X penicillin–streptomycin for at least 24 h. Cells were then plated on a 96-well polystyrene plate (Greiner, 655090) at about 1500 cells/well with the same stripped-serum DMEM. After about 3 days, the media was changed for stripped-serum DMEM containing 100 nM dexamethasone (MilliporeSigma) for 1, 2, or 3 h. The untreated cell media was replaced with media without dexamethasone. Dexamethasone treatment was stopped via fixation with 4% PFA in PBS. Cells were permeabilized in 0.1% Triton-X for 15 min at room temperature. We then blocked with 3% bovine serum albumin (BSA) at room temperature for 1 h. Cells were incubated with the anti-GR antibody G-5 (Santa Cruz Biotechnology, sc-393232) at a 1:500 dilution in 3% BSA overnight at 4ºC. This was followed by incubation with a goat anti-mouse antibody conjugated to Alexa Fluor 488 (Invitrogen, A-11001) in 3% BSA at a 1:1000 dilution for 1 h at room temperature. Nuclei staining was done using a 1:10,000 dilution of Hoechst in PBS for 15 min at room temperature. Between each step, two washes were done in each well using 100 μL PBS per wash. Images were obtained on a Nikon TiE microscope using a 10 × 0.4 numerical aperture objective. Nuclear GR signal was quantified from a nuclear mask, which was generated using Otsu’s method on cells stained for Hoechst. The cytoplasmic signal was extracted using a four-pixel-wide cytoplasmic ring dilated from the nuclear mask. The regionprops function in MATLAB was used to quantify the mean signal for both the nucleus and cytoplasm of each cell using the binary masks. The mean nuclear and cytoplasmic signal for each imaged cell was used to compare GR expression between cell lines or to calculate the nuclear fraction of total signal (the ratio of nuclear signal to the sum of nuclear and cytoplasmic signals).

### RNA immunoprecipitation of GR-HaloTag

Protocol was adapted from Holmes et al. with HaloTag-specific adaptations from Gu et al.^30,75^. U2OS cells stably expressing wt GR-HaloTag were grown to 10–15 million cells on 15 cm dishes in stripped-serum DMEM. Media was replaced with stripped-serum DMEM containing 100 nM dexamethasone for the treated samples or 0.01% ethanol (volume equivalent) for untreated samples. After 3 h of treatment, media was replaced with 13 mL ice cold 1 X PBS and the dish was subjected to 400 mJ/cm^2^ of 254 nm UV radiation (Stratalinker 1800). Cells were then harvested by scraping and transferred to a 15 mL conical tube. Cells were pelleted at 4 °C, 500×*g* for 5 min. Pellet was then washed in 1 mL ice cold 1 X PBS and pelleted at 4 °C, 4000×*g* for 75 s. PBS was aspirated and cell pellet was flash frozen in liquid nitrogen. Pellets were stored at − 70 °C.

Cell pellets were thawed and resuspended in 500 mL cold lysis buffer (50 mM Tris pH 8.0 at 4 °C, 150 mM NaCl, 0.1% SDS, 1% Triton X-100, 5 mM EDTA, 0.5% sodium deoxycholate, 1 mM DTT, 1 X protease inhibitor cocktail (Promega, G652A), and 100 U/mL RNaseOUT (Invitrogen)). Care must be taken to use protease inhibitor cocktails without AEBSF to avoid inhibition of HaloTag activity. Resuspension was rested on ice for 10 min and then cells were lysed with sonication (Bioruptor UCD-200) in an ice bath on high power, 30 s on/off for 10 min. Cell lysate was centrifuged at 16,100×*g* at 4 °C for 10 min. Supernatant was diluted with 500 μL of dilution buffer (50 mM Tris pH 8.0 at 4 °C, 150 mM NaCl, 5 mM EDTA, 1 mM DTT, 1 X protease inhibitor cocktail (Promega, G652A), and 100 U/mL RNaseOUT). 50 μL of the diluted supernatant was aliquoted as the input sample. 100 μL Magne HaloTag beads (Promega, G7281) were then washed in 200 μL dilution buffer. Diluted supernatant was added to the beads and rotated at 4 °C for 2 h. After bead incubation, supernatant was discarded and beads were washed 3 times with 200 μL high-salt PBST wash buffer (1 X PBS, 500 mM NaCl, 0.1% Triton X-100, 1 mM DTT, 100 U/mL RNaseOUT) and 2 times with 200 μL PBST wash buffer (1 X PBS, 0.1% Triton X-100, 1 mM DTT, 100 U/mL RNaseOUT). Beads were then washed three times with 200 μL 8 M urea with 3-min incubations for each. Then beads were washed three times with 200 μL SDS wash buffer (10% SDS, 50 mM Tris pH 8.0 at 4 °C, 1 mM EDTA, 1 mM DTT, 0.1% Triton X-100) with 3-min incubations for each. To wash out remaining denaturing washes, beads were again washed 3 times with 200 μL PBST wash buffer. Supernatant was removed and beads were stored overnight at − 20 °C. The purity of the pulldown was assessed using SDS-PAGE followed by silver stain and a parallel western blot using anti-GR antibody G-5 (Santa Cruz Biotechnology, sc-393232) as the primary antibody at a 1:100 dilution in TBS-T with 3% BSA. The secondary antibody was an HRP-conjugated rabbit anti-mouse antibody (Invitrogen, 31450) at a 1:5000 dilution in TBS-T with 3% BSA.

To isolate RNAs, beads were resuspended in 50 μL of nuclease-free water and 33 μL of 3 X reverse-crosslinking buffer (3 X PBS, 6% N-lauroyl sarcosine, 30 mM EDTA, 15 mM DTT, 2424 U/mL RNaseOUT). The same volume of 3 X reverse-crosslinking buffer was added to the input sample. 20 μL proteinase K (New England Biolabs) was then added to each sample. Samples were heated at 42 °C and 53 °C for one hour each with agitation every 10 min. Each sample was then mixed with 1 mL TRIzol (Invitrogen) and 200 μL chloroform followed by 20 s of vortex. Samples were centrifuged at 16,000×*g* for 15 min at 4 °C and the aqueous layer was transferred to a new tube. Each sample aqueous layer was then mixed with 500 μL isopropanol and 1 μL GlycoBlue (Invitrogen) and incubated at 4 °C for 10 min. Samples were centrifuged at 12,000×*g* for 10 min at 4 °C to pellet, supernatant was removed, and pellets were allowed to air dry for 5 min. Pellets were then resuspended in 85 μL nuclease-free water, 10 μL of 10 X DNase I reaction buffer, and 1 μL DNase I (New England Biolabs) and incubated at room temperature for 15 min. RNA was cleaned using the RNeasy MinElute Cleanup kit (Qiagen) with the quick start protocol. RNA was eluted in 14 μL nuclease-free water and frozen at − 20 °C.

RNA libraries were prepared using the KAPA RNA HyperPrep kit with RiboErase (Roche). For input samples, 1000 ng total RNA was used as input. For IP samples, 10 μL of sample was used due to low yield. Dual-index adapters including unique molecular identifiers were used to avoid PCR duplicates (IDT). For IP samples, 200 nM adapter stocks were used to avoid adapter contamination. Input sample libraries were PCR amplified for 9 cycles and IP sample libraries were amplified for 17 cycles. Samples were then sequenced on an Illumina NovaSEQ 6000 for 2 × 150 bp paired-end reads at the University of Colorado School of Medicine Genomics and Microarray Core Facility. Samples were sequenced to 18–24 million reads.

### 4-thiouridine (4sU) labeling and RNA isolation

Labeling was performed using a protocol adapted by the Goodrich-Kugel Lab (CU-Boulder) from Garibaldi et al.^81^. Briefly, each cell line was grown to about 80% confluence on 10 cm dishes in stripped-serum DMEM. Media was replaced with stripped-serum DMEM containing 100 nM dexamethasone for the treated samples or 0.01% ethanol (volume equivalent) for untreated samples. During the final hour of each treatment, media was supplemented with 200 µM 4-thiouridine (4sU, MilliporeSigma) to label newly transcribed RNAs. The untreated sample received 4sU for 1 h as well. At the end of treatment, cells were lysed by adding 3 mL TRIzol (Invitrogen) directly to the dish and incubating for 5 min. Total RNA was purified from the aqueous TRIzol fraction using isopropanol precipitation.

### 4sU-labeled RNA biotinylation and pulldown

Biotinylation was performed using a protocol adapted by the Goodrich-Kugel Lab (CU-Boulder) from Garibaldi et al.^81^. To biotinylate 4sU-labeled RNAs, we mixed 50–70 μg of total RNA with the biotinylation reaction buffer containing 10 mM HEPES pH 7.5, 1 mM EDTA, and 0.1 mg/mL biotin-MTSEA-XX in dimethylformamide (Biotium). Reaction was carried out for 30 min in the dark at room temperature and was followed by phenol–chloroform extraction and isopropanol precipitation. Streptavidin pulldown of biotinylated RNAs was performed using the μMACS streptavidin kit (Miltenyi Biotec) and RNAs were eluted with 100 mM DTT. Biotinylated RNAs were then precipitated in ethanol, resuspended in water, and frozen at − 20 °C. Typical yields were 2% of total RNA based on Qubit quantification. Biotinylation and pulldown were done in sample replicate pairs to reduce variability from the pulldown. The quality of purified RNA was assessed using a TapeStation (Agilent).

### Biotinylated RNA library preparation and sequencing

RNA libraries were prepared using the KAPA RNA HyperPrep kit with RiboErase (Roche). 400 ng of biotinylated RNA was used as input. Dual-index adapters including unique molecular identifiers were used to avoid PCR duplicates (IDT). Libraries were PCR amplified for 8 cycles and sequenced on an Illumina NovaSEQ 6000 for 2 × 150 bp paired-end reads at the University of Colorado School of Medicine Genomics and Microarray Core Facility. Samples were sequenced to 24–36 million reads.

### 4sU-seq analysis

Reads were tagged with UMIs using UMI-tools (v1.1.2)^82^. Reads were then trimmed using Trim Galore (v0.6.6) using the following parameters: -2colour 20, -paired^83^. Trimmed reads were aligned to the hg38 genome assembly using STAR (v2.7.3a) and deduplicated using UMI-tools (v1.1.2) using the following parameters: -paired, -unpaired-reads discard, -chimeric-pairs discard^84^. Deduplicated read coverage over exons was counted using featureCounts (Rsubread v2.0.1, R v4.0.3) and Gencode v39 gene annotations (https://www.gencodegenes.org/human/release_39.html)^38,85^.

Differential expression analysis was performed using DESeq2 (v1.34.0)^86^. For each cell line, differential expression results were obtained for each treatment time versus zero dexamethasone (0.01% ethanol). Results were also separately generated for each mutant versus the wild-type GR sample ignorant of treatment time. Time-dependent results were used to identify genes with stronger activation in the separation-of-function GR sample (“SoF Dex-dep.” genes). Mutation-dependent DESeq2 results were used to identify genes constitutively upregulated in the separation-of-function GR sample (“SoF Dex-ind.” genes). To select SoF Dex-dep. genes, bivariate outliers of log2 fold change between mutant samples at 3 h of dexamethasone treatment were identified using the minimum covariance determinant function provided by scikit-learn^87^. Outliers whose treatment fold changes were statistically significant (adjusted p-value < 0.05) in the SoF GR sample or both were then compared to a linear regression fit between the log2 fold changes of statistically significant genes from each sample to determine which genes exhibited the largest discrepancy in activation between SoF and Ctrl GR samples. Visual representations of this analysis can be found in Figs. S6 and 3A. The Enrichr web-based application was used for gene set enrichment and the Positional Gene Enrichment tool was used for determining enrichment of specific chromosomal regions^46,49^.

### RT-qPCR for measuring gene expression

Each cell line was grown to approximately 80% confluence in 6-well plates in stripped-serum DMEM. Media was replaced with stripped-serum DMEM containing 100 nM dexamethasone for the treated samples or 0.01% ethanol (volume equivalent) for untreated samples. At the end of treatment, cells were lysed by adding 500 μL TRIzol (Invitrogen) directly to the well and incubating for 5 min. Total RNA was purified from the aqueous TRIzol fraction using isopropanol precipitation. RT-qPCR was then carried out using the Luna Universal One-Step RT-qPCR Kit (New England Biolabs). 50 ng of total RNA was used in each reaction as the template and the forward and reverse primers for each gene added to a final concentration of 400 nM. Primer sequences can be found in Table S1. To calculate the relative expression of each gene, we first determined the ΔC_q_ values for each gene compared to our control gene RPLP0 (C_q, gene_ – C_q, RPLP0_). We then calculated the ΔΔC_q_ values between time points (ΔC_q, 3 h_ – ΔC_q, 0_) or GR mutant samples (ΔC_q, mutant_ – ΔC_q, wt_). This value was used to determine relative expression (relative expression = 2^−ΔΔCq^).

### ChIP-seq analysis

The U2OS GR ChIP-seq datasets used were from GSE109383 and GSE163398^37^. The nf-core/chipseq pipeline was used for data processing and bedtools intersect was used to select GR ChIP peaks present in both datasets^39,88^. Bedtools reldist and window functions were used to compare our 4sU gene sets to GR ChIP peaks^39^. XSTREME from the MEME-suite was used for motif discovery and comparison using sequences from relevant ChIP peaks^89^.

## Supplementary Information

Supplementary Information.

## Data Availability

Scripts for data processing and analysis can be found on Github (https://github.com/nicklammer). Raw and processed 4sU-seq and RIP-seq data can be found on GEO (4sU: GSE216337; RIP: GSE217888).

## References

[1] McDowell IC (2018). Glucocorticoid receptor recruits to enhancers and drives activation by motif-directed binding. Genome Res..

[2] Kino T, Hurt DE, Ichijo T, Nader N, Chrousos GP (2010). Noncoding RNA Gas5 is a growth arrest- and starvation-associated repressor of the glucocorticoid receptor. Sci. Signal..

[3] Weikum ER, Knuesel MT, Ortlund EA, Yamamoto KR (2017). Glucocorticoid receptor control of transcription: Precision and plasticity via allostery. Nat. Rev. Mol. Cell Biol..

[4] Ramamoorthy S, Cidlowski JA (2013). Ligand-induced repression of the glucocorticoid receptor gene is mediated by an NCoR1 repression complex formed by long-range chromatin interactions with intragenic glucocorticoid response elements. Mol. Cell. Biol..

[5] Bothe M, Buschow R, Meijsing SH (2021). Glucocorticoid signaling induces transcriptional memory and universally reversible chromatin changes. Life Sci. Alliance.

[6] Kadmiel M, Cidlowski JA (2013). Glucocorticoid receptor signaling in health and disease. Trends Pharmacol. Sci..

[7] Lee S, Krüger BT, Ignatius A, Tuckermann J (2022). Distinct glucocorticoid receptor actions in bone homeostasis and bone diseases. Front. Endocrinol..

[8] Adcock IM, Ito K (2005). Glucocorticoid pathways in chronic obstructive pulmonary disease therapy. Proc. Am. Thorac. Soc..

[9] Horby P (2021). Dexamethasone in hospitalized patients with Covid-19. N. Engl. J. Med..

[10] Htun H, Barsony J, Renyi I, Gould DL, Hager GL (1996). Visualization of glucocorticoid receptor translocation and intranuclear organization in living cells with a green fluorescent protein chimera. Proc. Natl. Acad. Sci..

[11] Savory JGA (1999). Discrimination between NL1- and NL2-mediated nuclear localization of the glucocorticoid receptor. Mol. Cell. Biol..

[12] Reddy TE (2009). Genomic determination of the glucocorticoid response reveals unexpected mechanisms of gene regulation. Genome Res..

[13] Luisi BF (1991). Crystallographic analysis of the interaction of the glucocorticoid receptor with DNA. Nature.

[14] Meijsing SH (2009). DNA binding site sequence directs glucocorticoid receptor structure and activity. Science.

[15] Watson LC (2013). The glucocorticoid receptor dimer interface allosterically transmits sequence-specific DNA signals. Nat. Struct. Mol. Biol..

[16] Schiller BJ, Chodankar R, Watson LC, Stallcup MR, Yamamoto KR (2014). Glucocorticoid receptor binds half sites as a monomer and regulates specific target genes. Genome Biol..

[17] Ishmael FT (2011). The human glucocorticoid receptor as an RNA-binding protein: Global analysis of glucocorticoid receptor-associated transcripts and identification of a target RNA motif. J. Immunol..

[18] Cho H (2015). Glucocorticoid receptor interacts with PNRC2 in a ligand-dependent manner to recruit UPF1 for rapid mRNA degradation. Proc. Natl. Acad. Sci..

[19] Park OH (2016). Identification and molecular characterization of cellular factors required for glucocorticoid receptor-mediated mRNA decay. Genes Dev..

[20] Hudson WH (2014). Conserved sequence-specific lincRNA–steroid receptor interactions drive transcriptional repression and direct cell fate. Nat. Commun..

[21] Parsonnet NV, Lammer NC, Holmes ZE, Batey RT, Wuttke DS (2019). The glucocorticoid receptor DNA-binding domain recognizes RNA hairpin structures with high affinity. Nucleic Acids Res..

[22] Los GV (2008). HaloTag: A novel protein labeling technology for cell imaging and protein analysis. ACS Chem. Biol..

[23] Ding S (2005). Efficient transposition of the piggyBac (PB) transposon in mammalian cells and mice. Cell.

[24] Rogatsky I, Trowbridge JM, Garabedian MJ (1997). Glucocorticoid receptor-mediated cell cycle arrest is achieved through distinct cell-specific transcriptional regulatory mechanisms. Mol. Cell. Biol..

[25] Rogatsky I (2003). Target-specific utilization of transcriptional regulatory surfaces by the glucocorticoid receptor. Proc. Natl. Acad. Sci..

[26] Meijsing SH, Elbi C, Luecke HF, Hager GL, Yamamoto KR (2007). The ligand binding domain controls glucocorticoid receptor dynamics independent of ligand release. Mol. Cell. Biol..

[27] Tiwari M, Oasa S, Yamamoto J, Mikuni S, Kinjo M (2017). A quantitative study of internal and external interactions of homodimeric glucocorticoid receptor using fluorescence cross-correlation spectroscopy in a live cell. Sci. Rep..

[28] Pujols L (2001). Expression of the Human glucocorticoid receptor α and β isoforms in human respiratory epithelial cells and their regulation by dexamethasone. Am. J. Respir. Cell Mol. Biol..

[29] Stavreva DA (2019). Transcriptional bursting and co-bursting regulation by steroid hormone release pattern and transcription factor mobility. Mol. Cell.

[30] Gu J (2018). GoldCLIP: Gel-omitted Ligation-dependent CLIP. Genomics Proteomics Bioinformatics.

[31] Rutkowski AJ (2015). Widespread disruption of host transcription termination in HSV-1 infection. Nat. Commun..

[32] Schwalb B (2016). TT-seq maps the human transient transcriptome. Science.

[33] Kino T (2009). Glucocorticoid receptor (GR) β has intrinsic, GRα-independent transcriptional activity. Biochem. Biophys. Res. Commun..

[34] Lewis-Tuffin LJ, Jewell CM, Bienstock RJ, Collins JB, Cidlowski JA (2007). Human glucocorticoid receptor β binds RU-486 and is transcriptionally active. Mol. Cell. Biol..

[35] Hudson WH, Youn C, Ortlund EA (2013). The structural basis of direct glucocorticoid-mediated transrepression. Nat. Struct. Mol. Biol..

[36] Frank F, Okafor CD, Ortlund EA (2018). The first crystal structure of a DNA-free nuclear receptor DNA binding domain sheds light on DNA-driven allostery in the glucocorticoid receptor. Sci. Rep..

[37] Lee BH, Stallcup MR (2018). Different chromatin and DNA sequence characteristics define glucocorticoid receptor binding sites that are blocked or not blocked by coregulator Hic-5. PLoS ONE.

[38] Frankish A (2019). GENCODE reference annotation for the human and mouse genomes. Nucleic Acids Res..

[39] Quinlan AR, Hall IM (2010). BEDTools: A flexible suite of utilities for comparing genomic features. Bioinformatics.

[40] Lim H-W (2015). Genomic redistribution of GR monomers and dimers mediates transcriptional response to exogenous glucocorticoid in vivo. Genome Res..

[41] Johnson TA, Paakinaho V, Kim S, Hager GL, Presman DM (2021). Genome-wide binding potential and regulatory activity of the glucocorticoid receptor’s monomeric and dimeric forms. Nat. Commun..

[42] Kulakovskiy IV (2018). HOCOMOCO: Towards a complete collection of transcription factor binding models for human and mouse via large-scale ChIP-Seq analysis. Nucleic Acids Res..

[43] Le DD (2018). Comprehensive, high-resolution binding energy landscapes reveal context dependencies of transcription factor binding. Proc. Natl. Acad. Sci..

[44] Levo M (2015). Unraveling determinants of transcription factor binding outside the core binding site. Genome Res..

[45] Schöne S (2016). Sequences flanking the core-binding site modulate glucocorticoid receptor structure and activity. Nat. Commun..

[46] Chen EY (2013). Enrichr: Interactive and collaborative HTML5 gene list enrichment analysis tool. BMC Bioinform..

[47] Préfontaine GG (1999). Selective binding of steroid hormone receptors to octamer transcription factors determines transcriptional synergism at the mouse mammary tumor virus promoter. J. Biol. Chem..

[48] Préfontaine GG (1998). Recruitment of octamer transcription factors to DNA by glucocorticoid receptor. Mol. Cell. Biol..

[49] De Preter K, Barriot R, Speleman F, Vandesompele J, Moreau Y (2008). Positional gene enrichment analysis of gene sets for high-resolution identification of overrepresented chromosomal regions. Nucleic Acids Res..

[50] Wallace AD, Cidlowski JA (2001). Proteasome-mediated glucocorticoid receptor degradation restricts transcriptional signaling by glucocorticoids. J. Biol. Chem..

[51] Deroo BJ (2002). Proteasomal inhibition enhances glucocorticoid receptor transactivation and alters its subnuclear trafficking. Mol. Cell. Biol..

[52] Stavreva DA, Müller WG, Hager GL, Smith CL, McNally JG (2004). Rapid glucocorticoid receptor exchange at a promoter is coupled to transcription and regulated by chaperones and proteasomes. Mol. Cell. Biol..

[53] Kinyamu HK, Archer TK (2007). Proteasome activity modulates chromatin modifications and rna polymerase II phosphorylation to enhance glucocorticoid receptor-mediated transcription. Mol. Cell. Biol..

[54] Wallace AD, Cao Y, Chandramouleeswaran S, Cidlowski JA (2010). Lysine 419 targets human glucocorticoid receptor for proteasomal degradation. Steroids.

[55] Presman DM (2016). DNA binding triggers tetramerization of the glucocorticoid receptor in live cells. Proc. Natl. Acad. Sci..

[56] Nader N, Chrousos GP, Kino T (2009). Circadian rhythm transcription factor CLOCK regulates the transcriptional activity of the glucocorticoid receptor by acetylating its hinge region lysine cluster: Potential physiological implications. FASEB J..

[57] Fletcher TM (2000). Structure and dynamic properties of a glucocorticoid receptor-induced chromatin transition. Mol. Cell. Biol..

[58] Carrigan A (2007). An active nuclear retention signal in the glucocorticoid receptor functions as a strong inducer of transcriptional activation. J. Biol. Chem..

[59] Vandevyver S, Dejager L, Libert C (2012). On the trail of the glucocorticoid receptor: Into the nucleus and back. Traffic.

[60] Galigniana MD, Echeverría PC, Erlejman AG, Piwien-Pilipuk G (2010). Role of molecular chaperones and TPR-domain proteins in the cytoplasmic transport of steroid receptors and their passage through the nuclear pore. Nucleus.

[61] Mazaira GI, Echeverria PC, Galigniana MD (2020). Nucleocytoplasmic shuttling of the glucocorticoid receptor is influenced by tetratricopeptide repeat-containing proteins. J. Cell Sci..

[62] Matthews L (2011). Cell cycle phase regulates glucocorticoid receptor function. PLoS ONE.

[63] Quinodoz SA (2021). RNA promotes the formation of spatial compartments in the nucleus. Cell.

[64] Jachowicz JW (2022). Xist spatially amplifies SHARP/SPEN recruitment to balance chromosome-wide silencing and specificity to the X chromosome. Nat. Struct. Mol. Biol..

[65] Ukmar-Godec T (2019). Lysine/RNA-interactions drive and regulate biomolecular condensation. Nat. Commun..

[66] Arenas A (2020). Lysine acetylation regulates the RNA binding, subcellular localization and inclusion formation of FUS. Hum. Mol. Genet..

[67] Gal J (2019). The acetylation of lysine-376 of G3BP1 regulates RNA binding and stress granule dynamics. Mol. Cell. Biol..

[68] Steiner HR, Lammer NC, Batey RT, Wuttke DS (2022). An extended DNA binding domain of the estrogen receptor alpha directly interacts with RNAs in vitro. Biochemistry.

[69] Yang M (2020). Enhancer RNAs mediate estrogen-induced decommissioning of selective enhancers by recruiting ERα and its cofactor. Cell Rep..

[70] Xu Y (2021). ERα is an RNA-binding protein sustaining tumor cell survival and drug resistance. Cell.

[71] Jeon Y, Lee JT (2011). YY1 Tethers Xist RNA to the inactive X nucleation center. Cell.

[72] Sigova AA (2015). Transcription factor trapping by RNA in gene regulatory elements. Science.

[73] Davis BN, Hilyard AC, Nguyen PH, Lagna G, Hata A (2010). Smad proteins bind a conserved RNA sequence to promote microRNA maturation by Drosha. Mol. Cell.

[74] Dickey TH, Pyle AM (2017). The SMAD3 transcription factor binds complex RNA structures with high affinity. Nucleic Acids Res..

[75] Holmes ZE (2020). The Sox2 transcription factor binds RNA. Nat. Commun..

[76] Pelham HR, Brown DD (1980). A specific transcription factor that can bind either the 5S RNA gene or 5S RNA. Proc. Natl. Acad. Sci. USA..

[77] Setzer DR, Menezes SR, Rio SD, Hung VS, Subramanyan G (1996). Functional interactions between the zinc fingers of Xenopus transcription factor IIIA during 5S rRNA binding. RNA.

[78] Niessing D (2000). Homeodomain position 54 specifies transcriptional versus translational control by bicoid. Mol. Cell.

[79] He C (2016). High-resolution mapping of RNA-binding regions in the nuclear proteome of embryonic stem cells. Mol. Cell.

[80] Castello A (2016). Comprehensive identification of RNA-binding domains in human cells. Mol. Cell.

[81] Garibaldi A, Carranza F, Hertel KJ (2017). Isolation of newly transcribed RNA using the metabolic label 4-thiouridine. Methods Mol. Biol. Clifton NJ.

[82] Smith T, Heger A, Sudbery I (2017). UMI-tools: Modeling sequencing errors in Unique Molecular Identifiers to improve quantification accuracy. Genome Res..

[83] Krueger, F. Trim Galore! https://www.bioinformatics.babraham.ac.uk/projects/trim_galore/.

[84] Dobin A (2013). STAR: Ultrafast universal RNA-seq aligner. Bioinformatics.

[85] Liao Y, Smyth GK, Shi W (2019). The R package Rsubread is easier, faster, cheaper and better for alignment and quantification of RNA sequencing reads. Nucleic Acids Res..

[86] Love MI, Huber W, Anders S (2014). Moderated estimation of fold change and dispersion for RNA-seq data with DESeq2. Genome Biol..

[87] Pedregosa F (2011). Scikit-learn: Machine learning in python. J. Mach. Learn. Res..

[88] Ewels PA (2020). The nf-core framework for community-curated bioinformatics pipelines. Nat. Biotechnol..

[89] Bailey TL, Johnson J, Grant CE, Noble WS (2015). The MEME suite. Nucleic Acids Res..

