# Supplementary material for: RNA binding by the glucocorticoid receptor attenuates dexamethasone-induced gene activation
Source: Sci Rep. 2023 Jun 9;13:9385. doi: 10.1038/s41598-023-35549-y (PMC10251336; doi:10.1038/s41598-023-35549-y)
Supplement: Supplementary file 1 — Supplementary Information. [file 41598_2023_35549_MOESM1_ESM.docx]

**Supplementary Information**

**RNA binding by the Glucocorticoid Receptor Attenuates Dexamethasone-Induced Gene Activation**

Nickolaus C. Lammer, Humza M. Ashraf, Daniella A. Ugay, Sabrina L. Spencer, Mary A. Allen, Robert T. Batey, Deborah S. Wuttke

Contents

Figure S1: Silver stain and western blot of GR-HaloTag pulldown (RIP)

Figure S2: PCA of gene coverage from each replicate plotted using DESeq2

Figure S3: UpSet plots comparing differential expression from dexamethasone treatment

Figure S4: Heatmap of gene abundance for genes differentially expressed in wt GR cells

Figure S5: Pairwise fold change scatter plots for differential expression after dexamethasone treatment

Figure S6: Pairwise fold change scatter plot outliers

Figure S7: Representative gene tracks, normalized gene counts, and relative expression for SoF Dex-dep. genes

Figure S8: Heatmap of SoF Dex-dep. gene abundance

Figure S9: Genes downregulated in SoF GR cells relative to Ctrl and wt GR cells

Figure S10: Heatmap of gene abundance for activated genes downregulated in SoF (SoF 3h Rep.)

Figure S11: Representative gene tracks, normalized gene counts, and relative expression for SoF Dex-ind. genes

Figure S12: Heatmap of SoF Dex-ind. gene abundance

Figure S13: Heatmap of gene abundance for genes constitutively downregulated in SoF (SoF Const. Rep.)

Figure S14: Enrichr plot for SoF 3h Rep. and SoF Const. Rep. gene sets

Figure S15: Line plots of SoF Dex-dep. and SoF Dex-ind. gene abundance

Figure S16: Abundance of SoF Dex-dep. genes from GR-HaloTag RIP-seq

Figure S17: Alignment of select nuclear receptor DNA-binding domains with hinge regions

Table S1: RT-qPCR primers

| **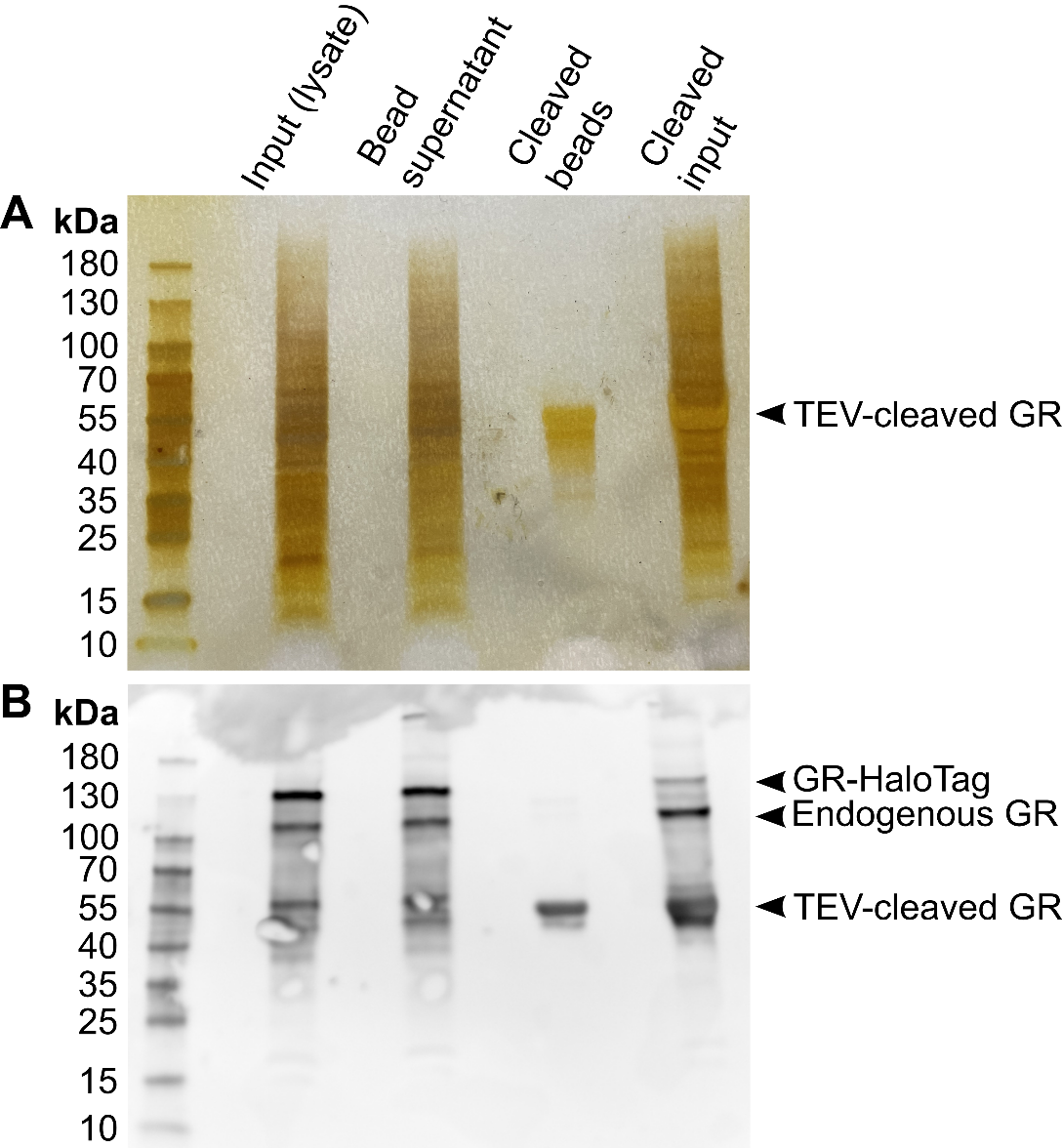** |
| --- |
| **Figure S1. Silver stain and western blot of GR-HaloTag pulldown (RIP).** (A and B) Silver stain (A) and western blot (B) showing the quality of the HaloTag pulldown used in the RIP experiment. Samples included are protein markers, input (cell lysate), the supernatant after incubation of the lysate with the HaloTag-capturing beads, bead sample after TEV cleavage, and input after TEV cleavage. TEV cleavage produces a product shorter than endogenous GR which required the inclusion of TEV-cleaved input. |

| **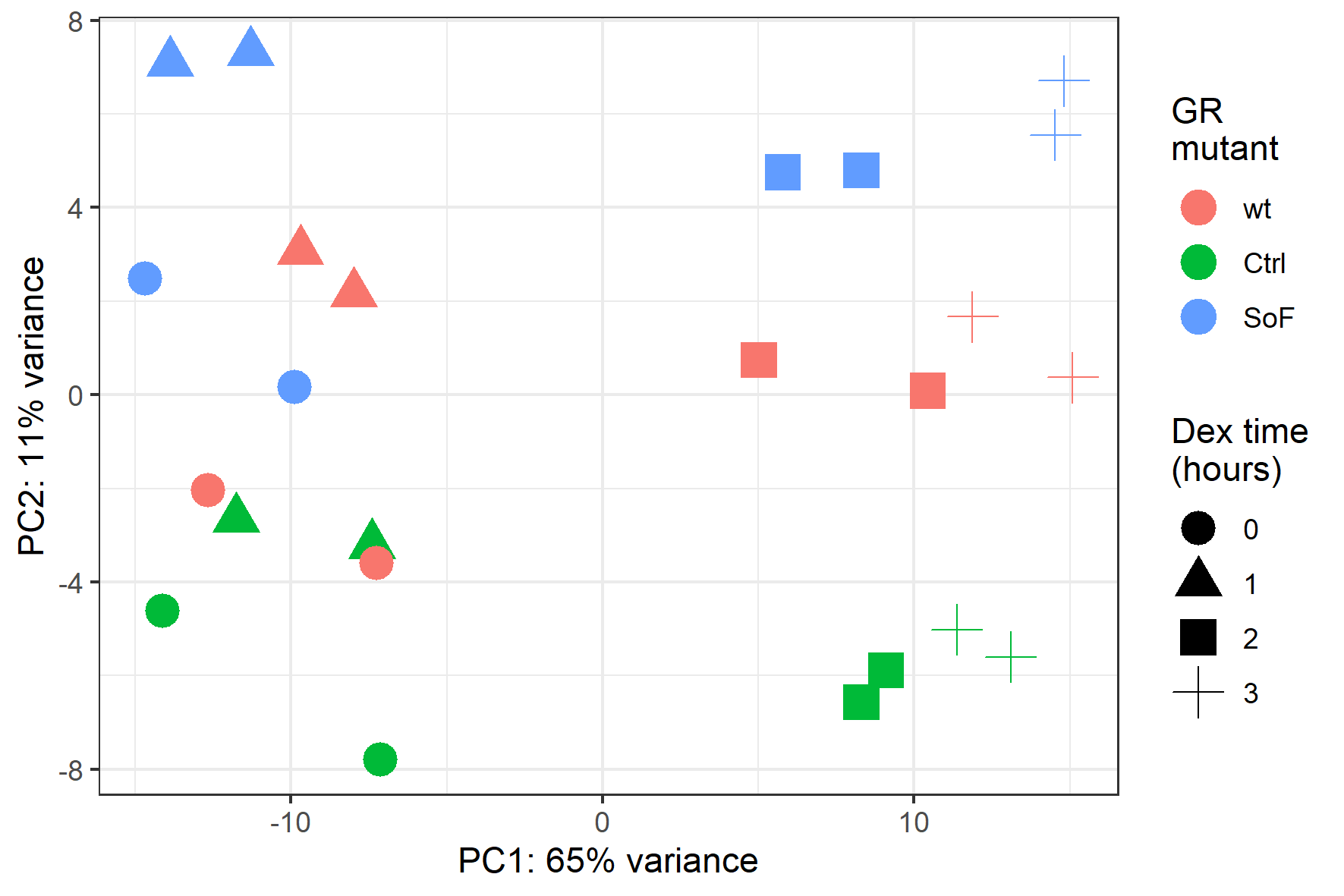** |
| --- |
| **Figure S2. PCA of gene coverage from each replicate plotted using DESeq2.** Regularized log transformed count data for each 4sU-seq replicate was used to make PCA plots in DESeq2. The PCA plot is grouped by GR mutant (colors) and treatment time (shapes). Dots with the same shape and color represent replicates (n = 2). |

| **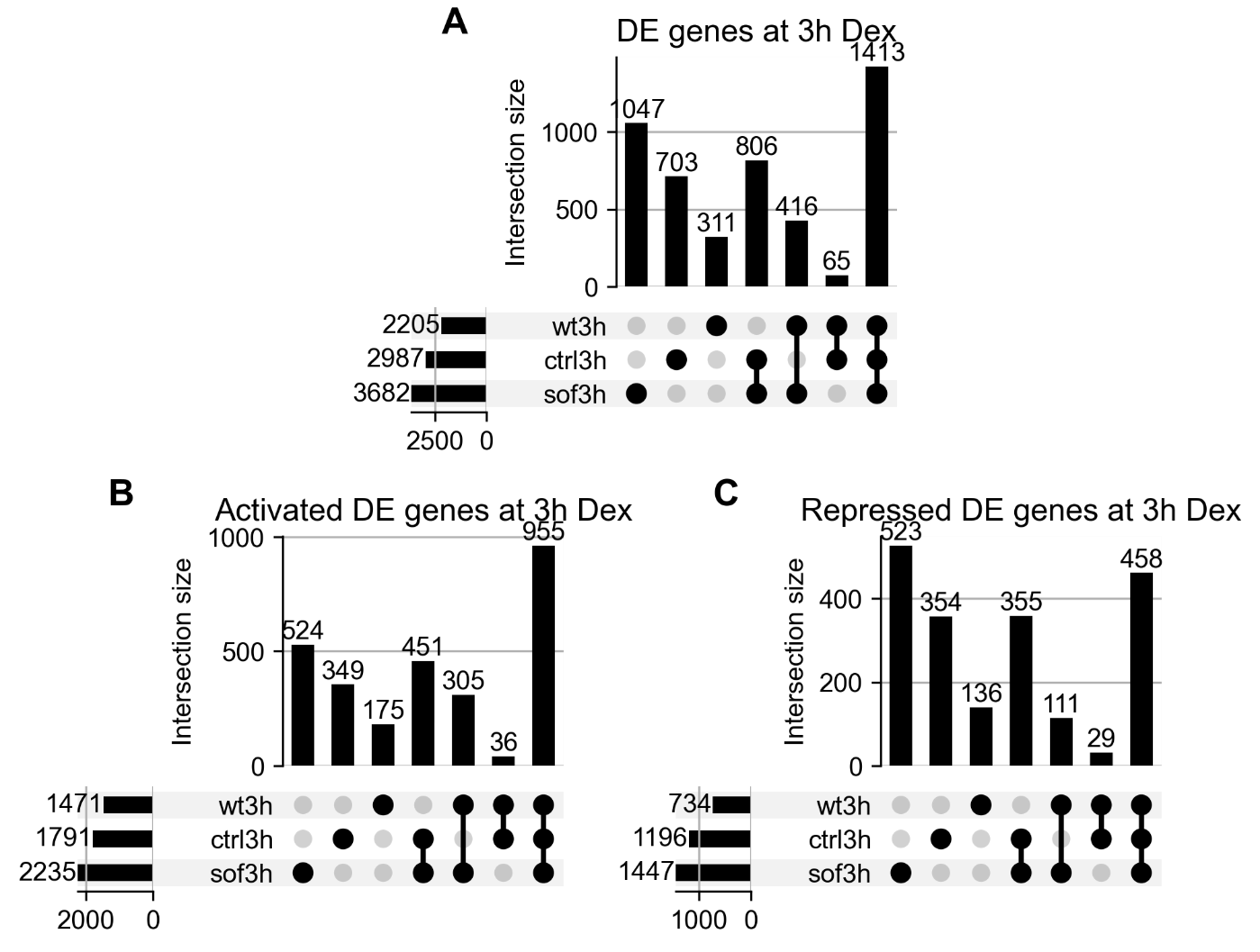** |
| --- |
| **Figure S3. UpSet plots comparing differential expression from dexamethasone treatment.** UpSet plots showing the overlap of genes differentially expressed after 3 hours of 100 nM dexamethasone treatment in each GR cell line. Differential expression is defined as having a fold change adjusted p-value < 0.05. Plots of all differentially expressed genes (A), activated genes (B), and repressed genes (C) and shown. |

| **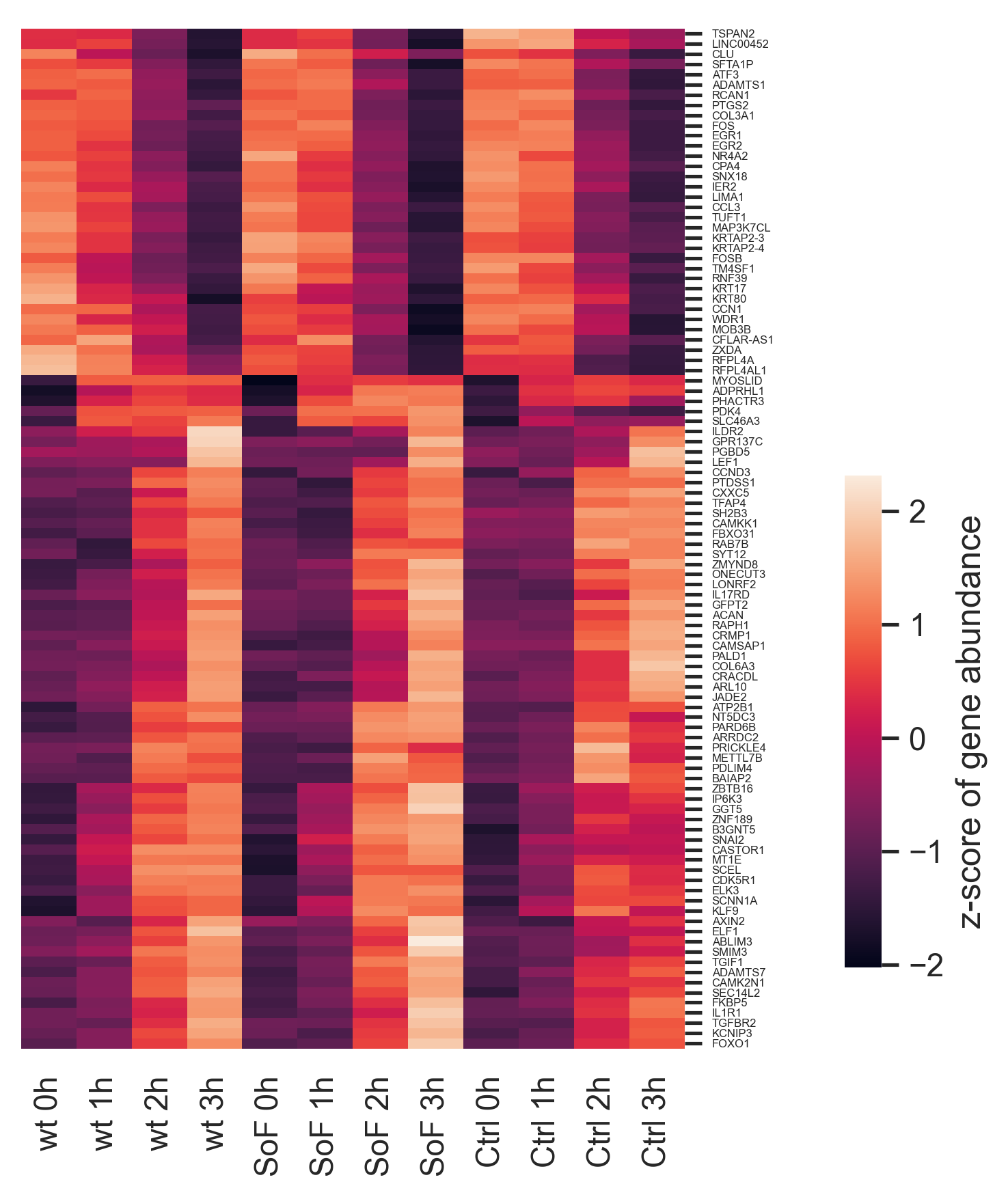** |
| --- |
| **Figure S4. Heatmap of gene abundance for genes differentially expressed in wt GR cells.** Z-score of gene abundance in each cell line and 100 nM dexamethasone time point for the 100 most differentially expressed genes in wt GR cells after 3 hours of dexamethasone treatment based on adjusted p-value. |

| **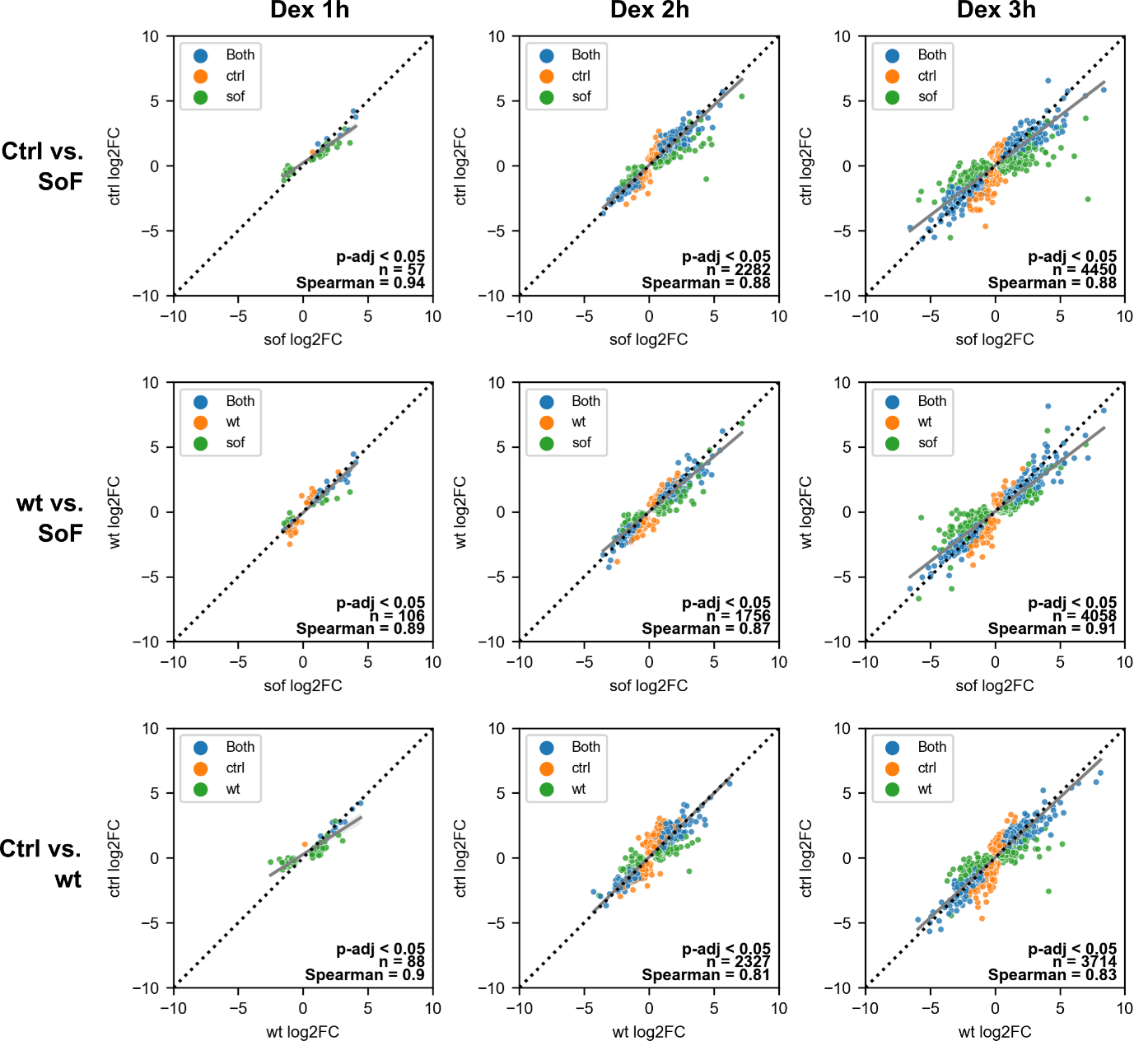** |
| --- |
| **Figure S5. Pairwise fold change scatter plots for differential expression after dexamethasone treatment.** Fold change scatter plots of sample pairs over the 100 nM dexamethasone time course. Each column is a different hour of the dexamethasone time course and each row is a different sample pair. Dot colors represent adjusted p-value of fold change < 0.05 in either or both samples shown. Gray line is the linear regression fit line and dashed line delineates equal x-y values. Spearman correlation coefficient and n total number of genes is listed. |

| **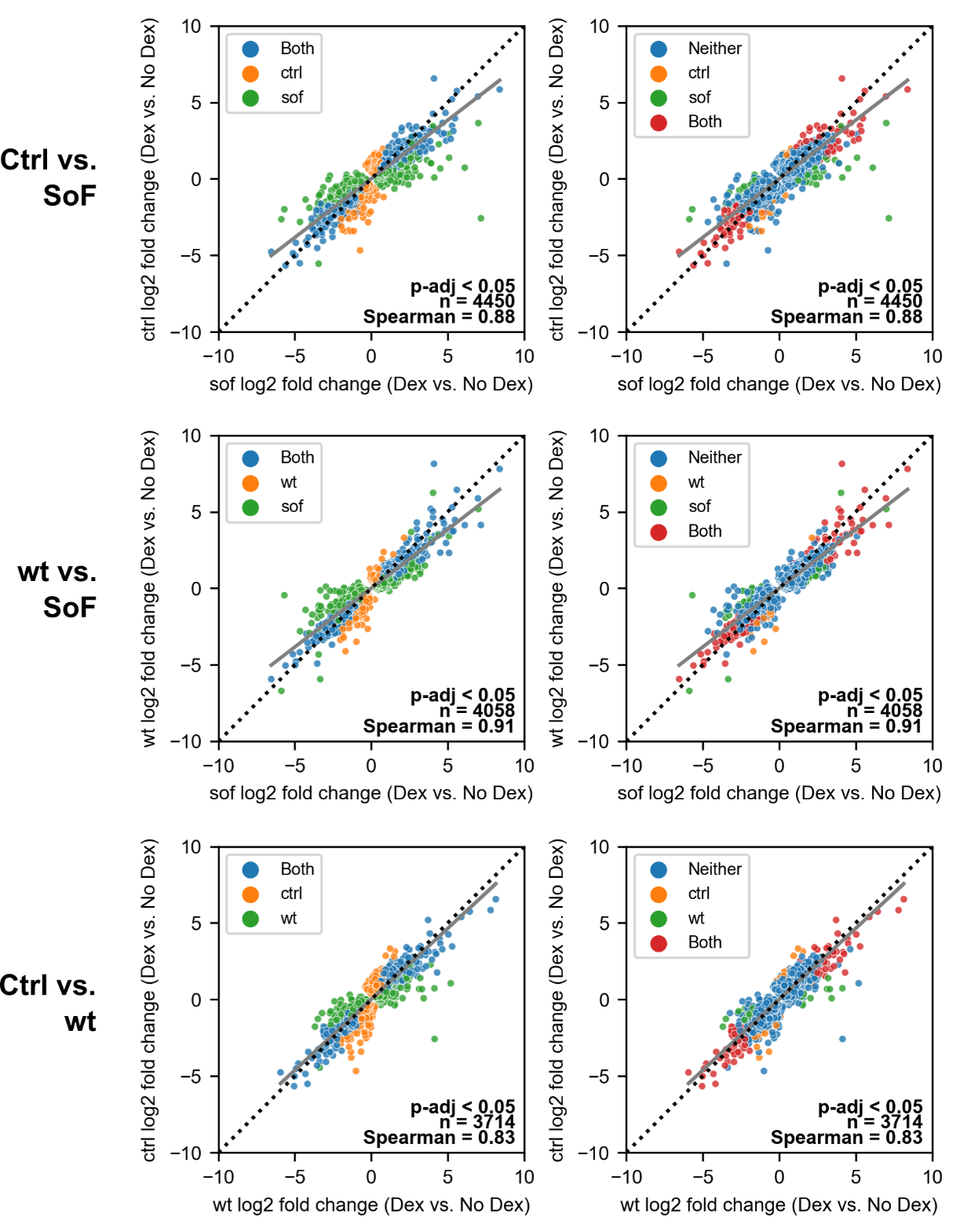** |
| --- |
| **Figure S6.** **Pairwise fold change scatter plot outliers.** Left column is the same fold change scatter plot from Figure S5 at 3 hours dexamethasone treatment. Right column shows the same scatter plot, but dot colors now indicate in which sample an outlier is statistically significant (adjusted p-value of fold change < 0.05). Minimum covariance determinant was used to estimate bivariate outliers from genes that were differentially expressed in either sample after passing an adjusted p-value threshold of 0.05. Gray line is the linear regression fit line and dashed line delineates equal x-y values. Spearman correlation coefficient and n total number of genes is listed. |

| **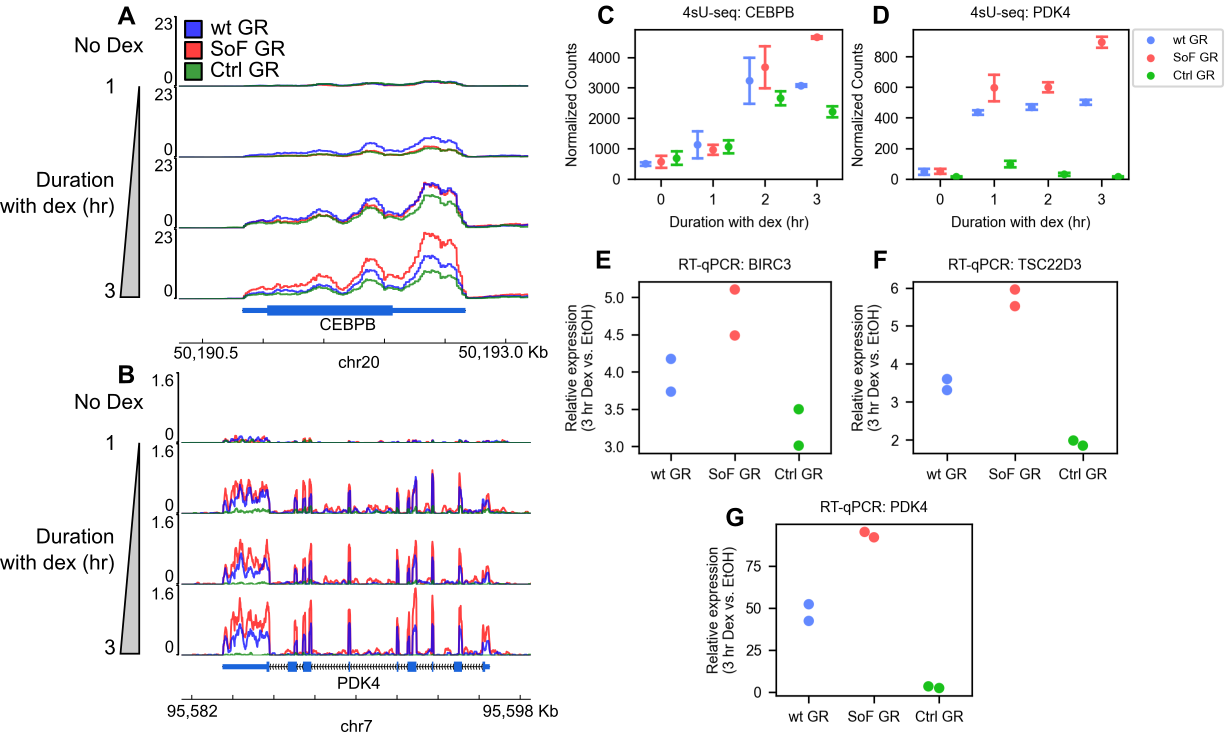** |
| --- |
| **Figure S7. Representative gene tracks, normalized gene counts, and relative expression for SoF Dex-dep. genes.** (A, B) Representative gene tracks from two SoF Dex-dep. genes: CEBPB (A) and PDK4 (B). Vertical progression corresponds to increasing 100 nM dexamethasone treatment time. Tracks display 4sU-seq reads normalized to transcripts per million plotted against chromosome coordinates. (C, D) Normalized gene counts generated by DESeq2 for CEBPB (C) and PDK4 (D) over the dexamethasone treatment time. (E, F, H) Expression of BIRC3 (E), TSC22D3 (F), and PDK4 (G) in each GR cell line after 3 hours of 100 nM dexamethasone treatment normalized to ethanol (vehicle) treatment as determined by RT-qPCR (n=2). Gene expression was determined in relation to RPLP0. Each of 2 replicates is shown. Error bars in C and D represent 95% confidence intervals. |

| **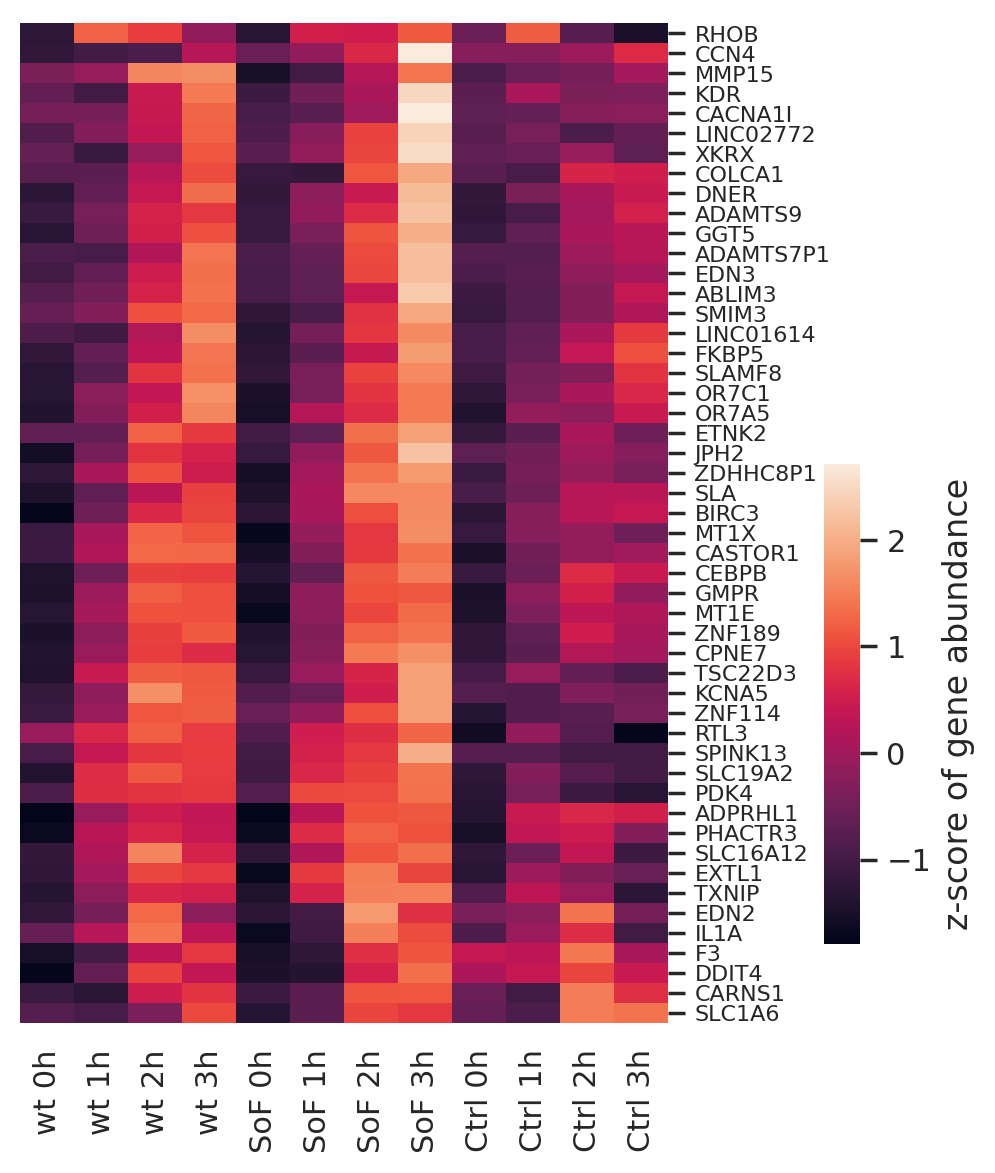** |
| --- |
| **Figure S8. Heatmap of SoF Dex-dep. gene abundance.** Z-score of gene abundance in each cell line and 100 nM dexamethasone time point for each gene in the SoF Dex-dep. gene set. Total size is 50 genes. |

| **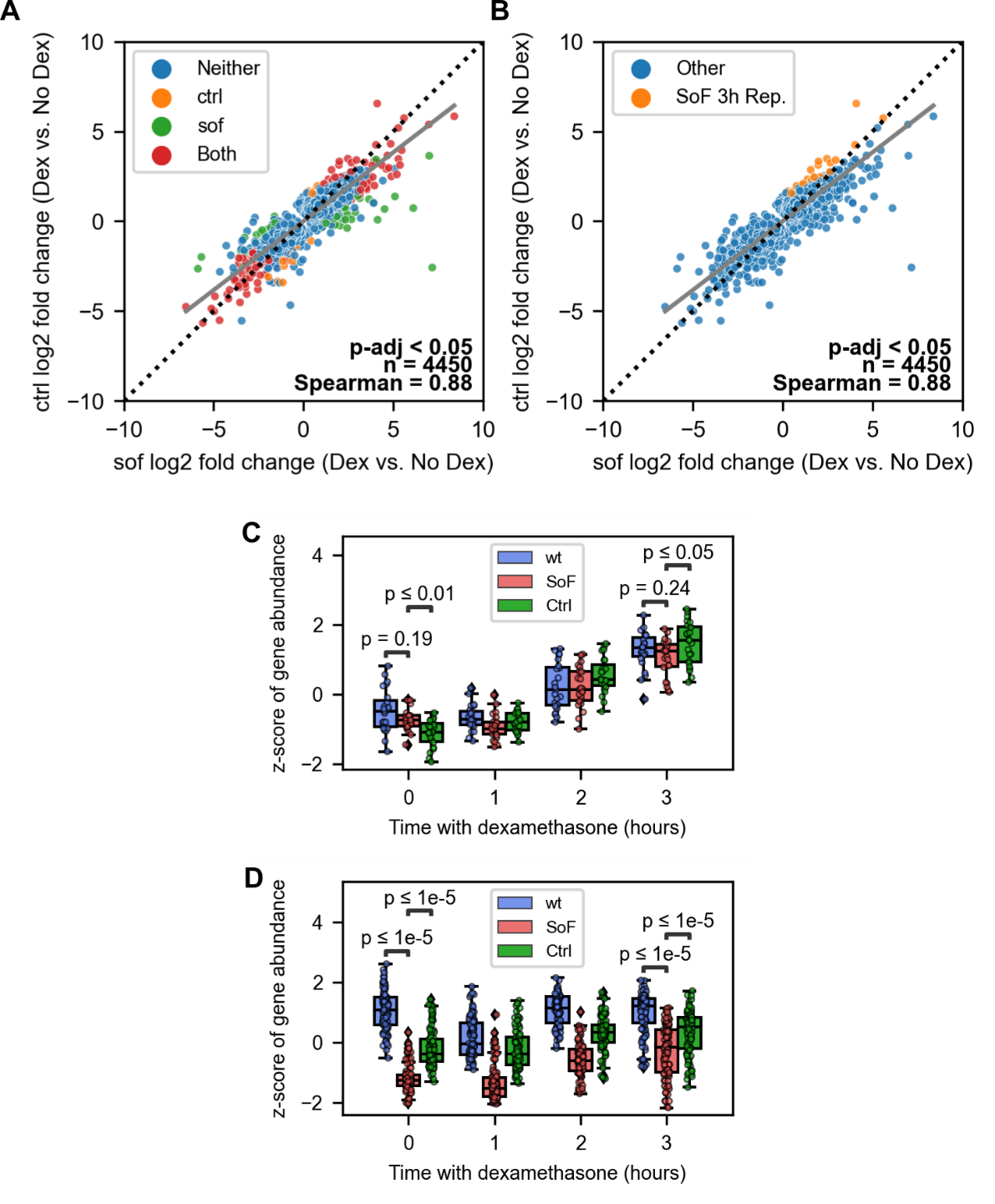** |
| --- |
| **Figure S9. Genes downregulated in SoF GR cells relative to Ctrl and wt GR cells.** (A, B) Fold change scatter plots showing outlier genes at 3 h dexamethasone treatment for SoF vs. Ctrl GR as in Figure S6 (A) and genes selected as downregulated in SoF GR cells relative to Ctrl GR cells (SoF 3h Rep., B). (C, D) Box and dot plots showing the z-score of gene abundance for SoF 3h Rep. genes (C) or genes constitutively downregulated in SoF GR cells relative to wt GR cells (SoF Const. Rep., D) over the 100 nM dexamethasone time course. Abundance is blue for the wt GR cells, red for the SoF mutant, and green for the Ctrl mutant. Significance testing was performed using Mann-Whitney tests. |

| **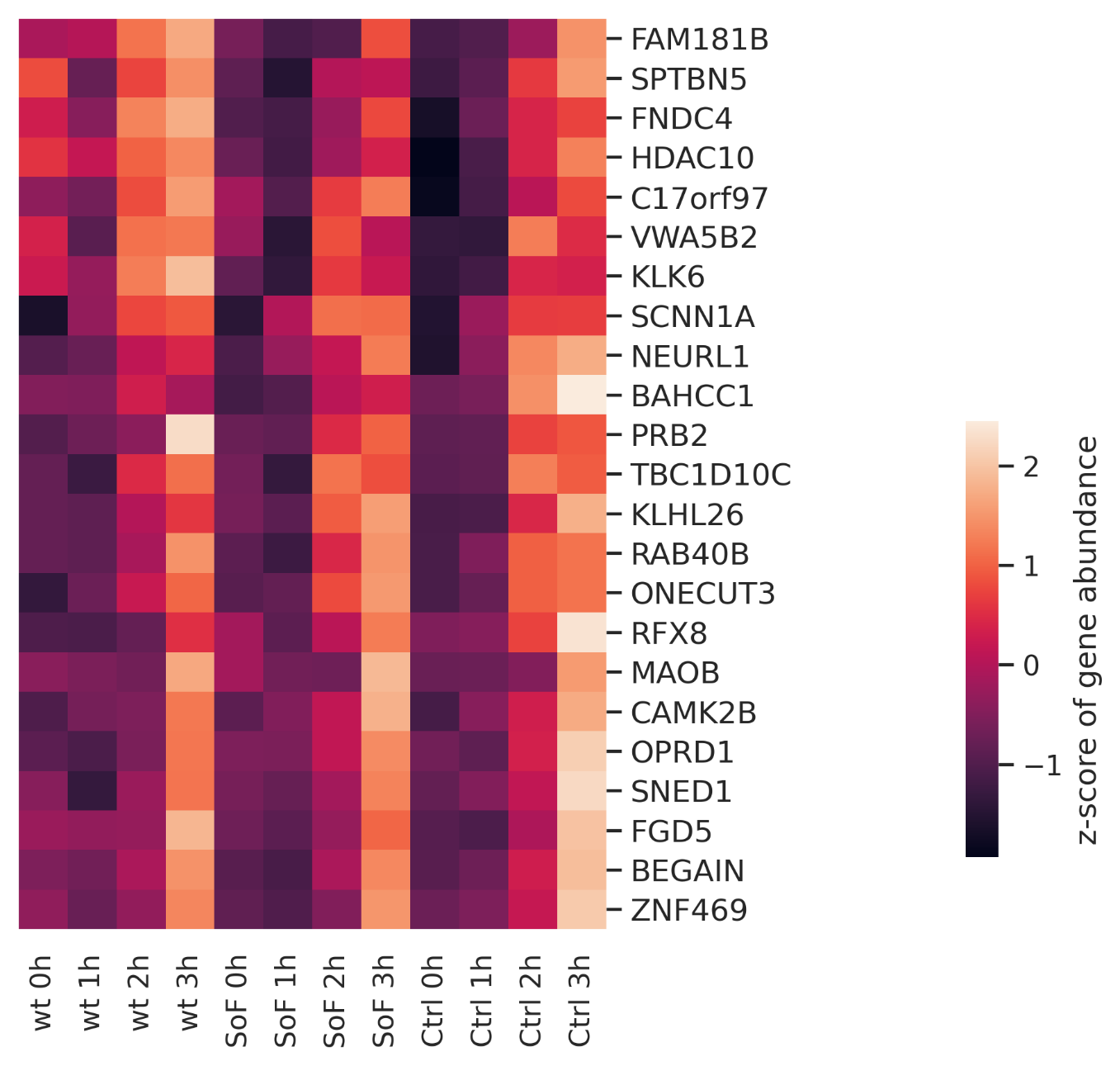** |
| --- |
| **Figure S10. Heatmap of gene abundance for activated genes downregulated in SoF (SoF 3h Rep.).** Z-score of gene abundance in each cell line and 100 nM dexamethasone time point for each gene in the set of genes downregulated at 3 h dexamethasone in SoF GR cells. Total size is 23 genes. |

| **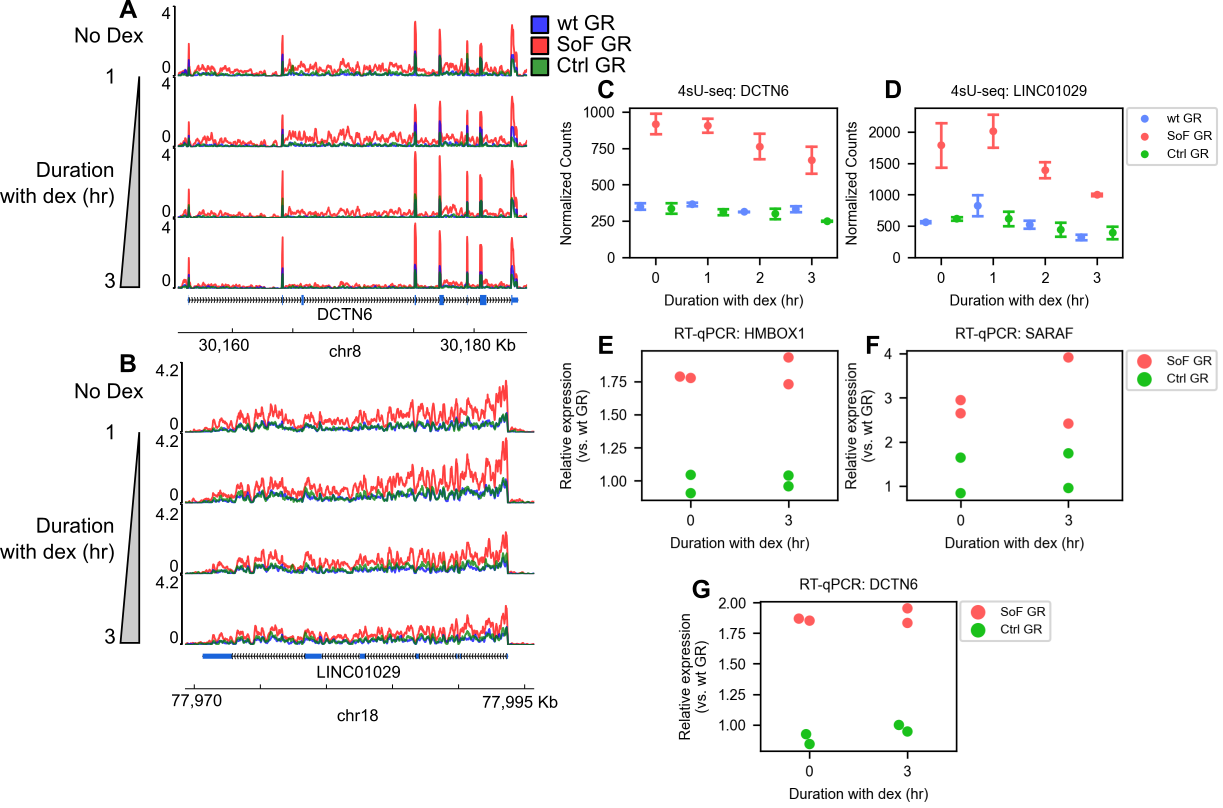** |
| --- |
| **Figure S11. Representative gene tracks, normalized gene counts, and relative expression for SoF Dex-ind. genes.** (A, B) Representative gene tracks from two SoF Dex-ind. genes: DCTN6 (A) and LINC01029 (B). Vertical progression corresponds to increasing 100 nM dexamethasone treatment time. Tracks display 4sU-seq reads normalized to transcripts per million plotted against chromosome coordinates. (C, D) Normalized gene counts generated by DESeq2 for DCTN6 (C) and LINC01029 (D) over the dexamethasone treatment time. (E, F, G) Expression of HMBOX1 (E), SARAF (F), and DCTN6 (G) in SoF and Ctrl GR cell lines with ethanol (vehicle) and 3 hours of 100 nM dexamethasone treatment normalized to wt GR cells as determined by RT-qPCR (n=2). Gene expression was determined in relation to RPLP0. Each of 2 replicates is shown. Error bars in C and D represent 95% confidence intervals. |

| **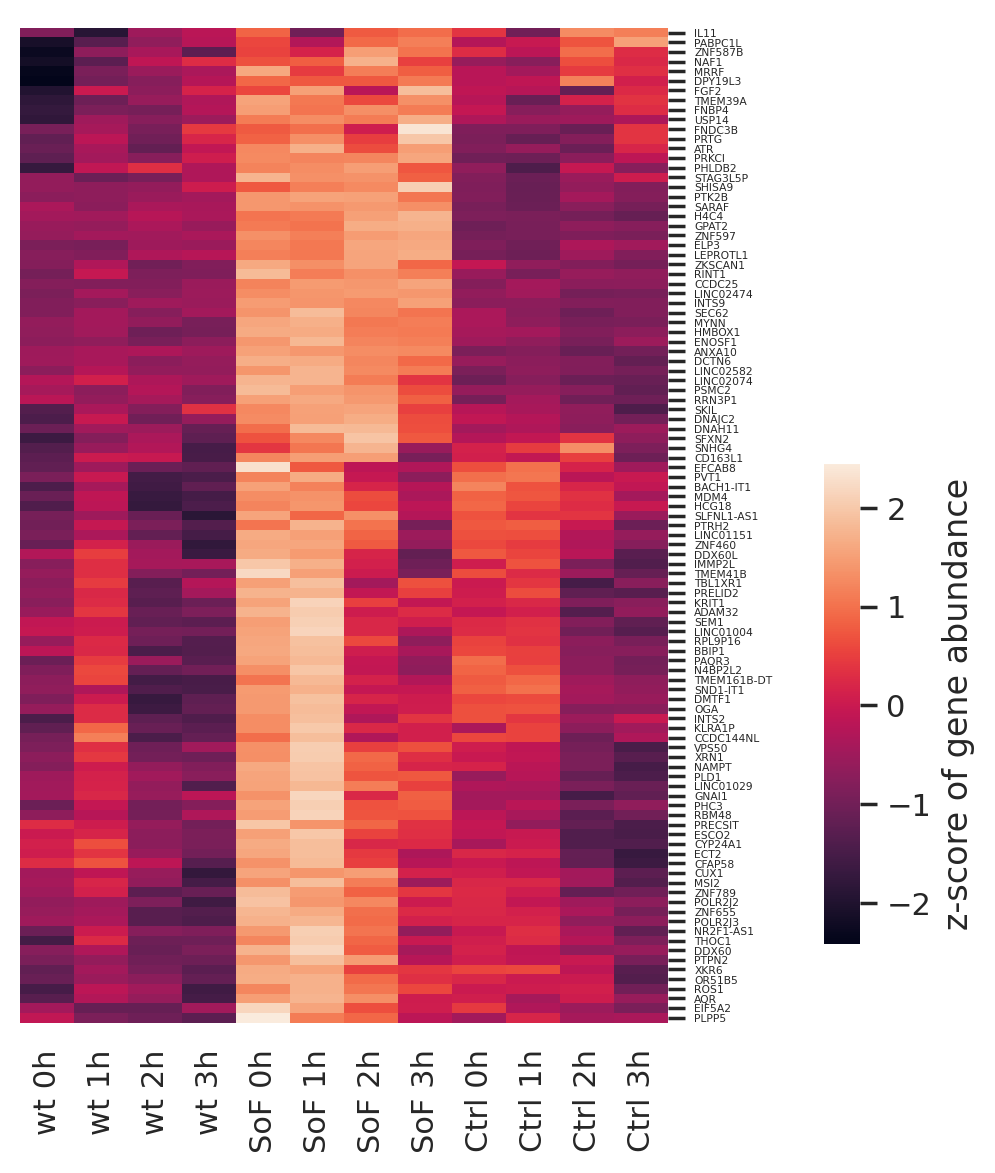** |
| --- |
| **Figure S12. Heatmap of SoF Dex-ind. gene abundance.** Z-score of gene abundance in each cell line and 100 nM dexamethasone time point for each gene in the SoF Dex-ind. gene set. Total size is 103 genes. |

| **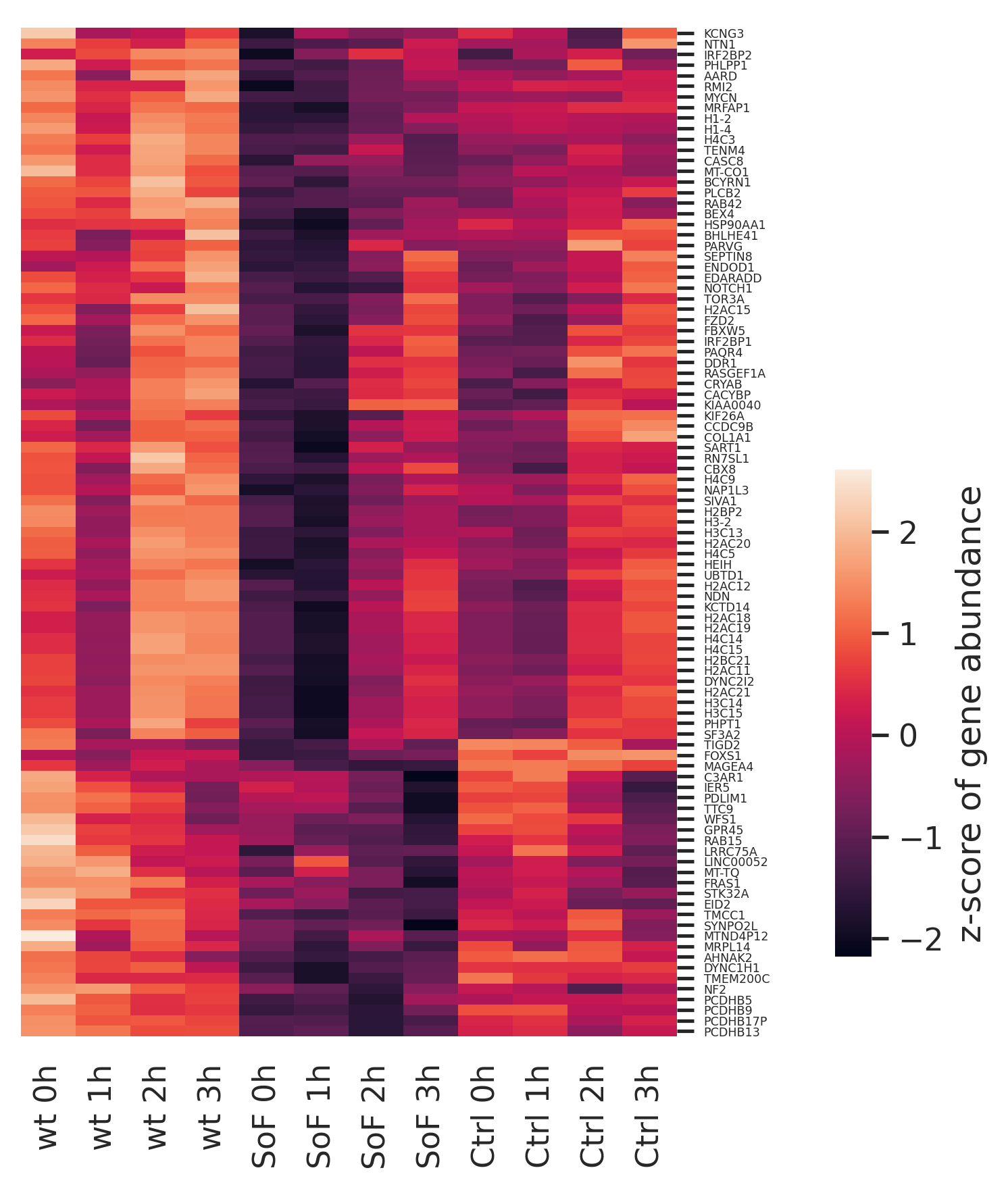** |
| --- |
| **Figure S13. Heatmap of gene abundance for genes constitutively downregulated in SoF (SoF Const. Rep.).** Z-score of gene abundance in each cell line and 100 nM dexamethasone time point for each gene in the set of genes constitutively downregulated relative to wt GR cells in SoF GR cells. Total size is 95 genes. |

| **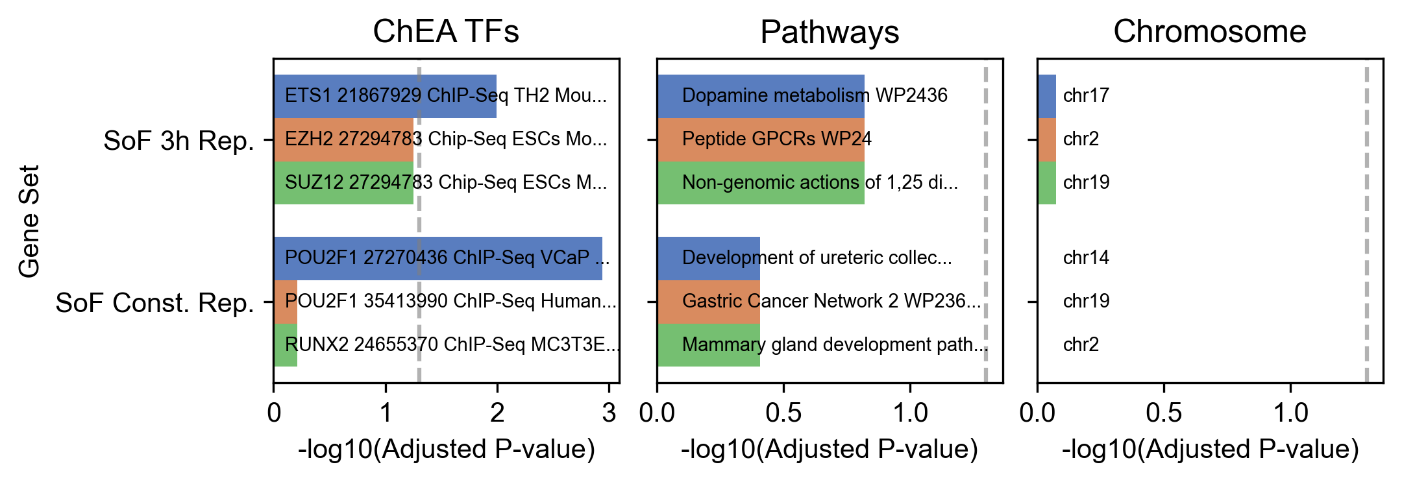** |
| --- |
| **Figure S14. Enrichr plot for SoF 3h Rep. and SoF Const. Rep. gene sets.** Enrichr gene set analysis with sets of interest for ChEA TF targets, WikiPathways, and chromosome^1^. Plotted are the top three terms in each category based on the adjusted p-value. The dashed gray line indicates an adjusted p-value = 0.05. |

| **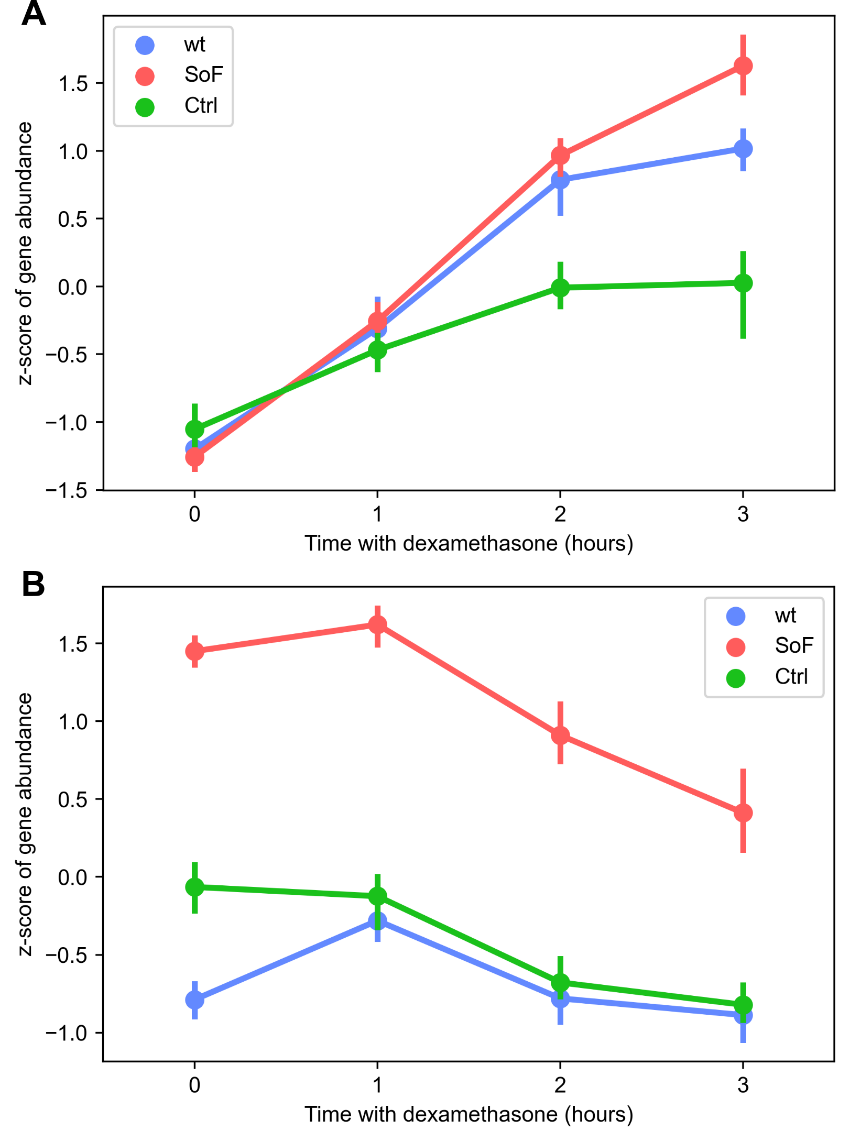** |
| --- |
| **Figure S15. Line plots of SoF Dex-dep. and SoF Dex-ind. gene abundance.** Line plots showing the z-score of gene abundance for the SoF Dex-dep. (A) and SoF Dex-ind. (B) genes over the 100 nM dexamethasone time course. The data is the same as found in Figures 3B (A) and 4A (B). Points represent the median at each time for the corresponding cell line. Error bars correspond to a 95% confidence interval. |

| **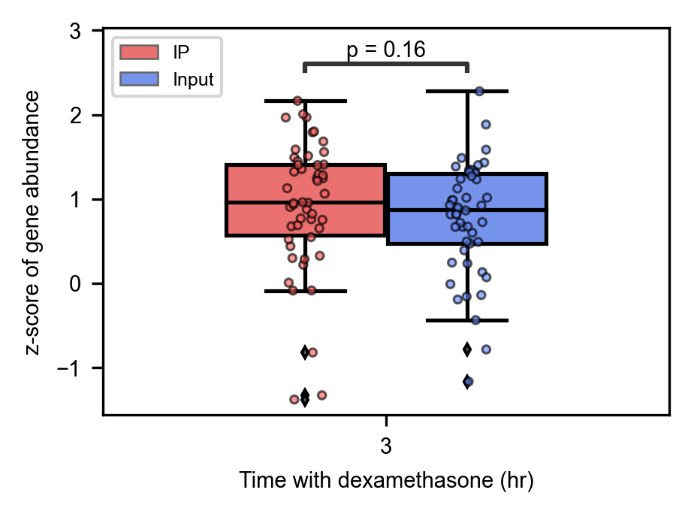** |
| --- |
| **Figure S16.** **Abundance of SoF Dex-dep. genes from GR-HaloTag RIP-seq.** Box and dot plot showing the z-score of gene abundance from GR-HaloTag RIP-seq for SoF Dex-dep. genes after 3 hours of 100 nM dexamethasone treatment. Abundance is red for the IP (GR-bound) sample and blue for the input sample. Significance testing was performed using a Mann-Whitney test. |
| **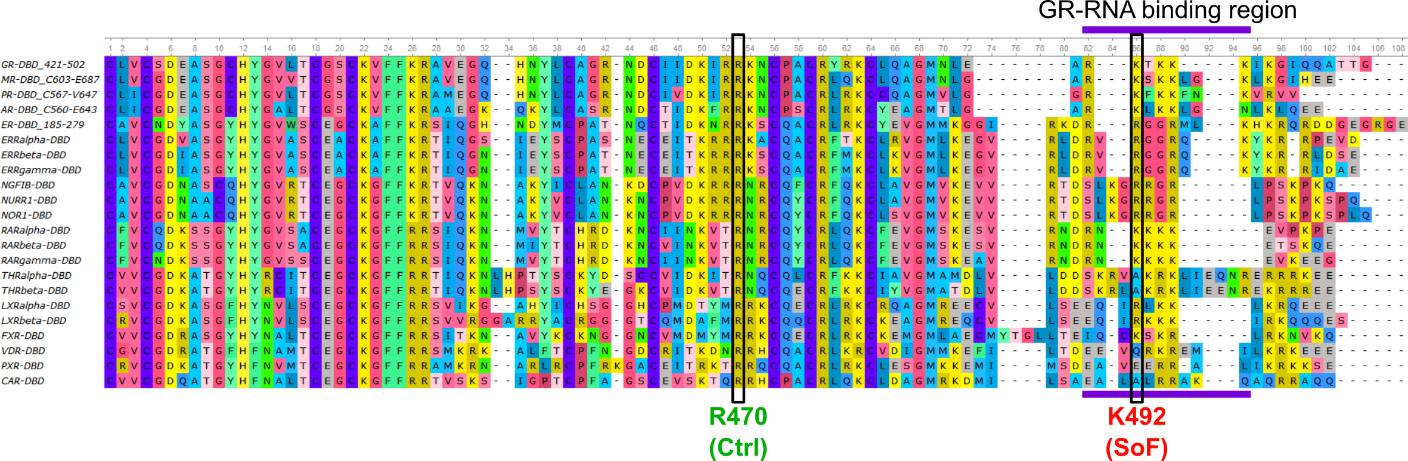** |
| **Figure S17. Alignment of select nuclear receptor DNA-binding domains with hinge regions.** DBDs with adjacent hinge regions were aligned using UGENE and MUSCLE alignment with sequences from RefSeq.^2–4^ Green bars indicate the region of GR-DBDext implicated in RNA binding. |

**Table S1. RT-qPCR primers**

| **Gene** | **Forward Primer (5’-3’)** | **Reverse Primer (5’-3’)** |
| --- | --- | --- |
| RPLP0 | CGTCCTCGTGGAAGTGACAT | TAGTTGGACTTCCAGGTCGC |
| BIRC3 | TCTGGGCAGCAGGTTTACAA | CCCGAGATTAGACTAAGTCCCTT |
| TSC22D3 | CCATGGACATCTTCAACAGC | TTGGCTCAATCTCTCCCATC |
| PDK4 | CTGGTGTATCCCAAGCAGGG | CAGGAAGCAGCACTGGTGTA |
| HMBOX1 | CGGAGAGAACTCAGCGAGG | CCAGCAAAACCACTGGAAAGG |
| SARAF | GCAGTGACGTAGGGTTGGC | CCGAACCTGGGTGCGGTAG |
| DCTN6 | TGGCTGTTATTCCCAAGCCAT | TTAGGTTGCAACAAGCCCCA |

**REFERENCES**

1. Chen, E. Y. *et al.* Enrichr: interactive and collaborative HTML5 gene list enrichment analysis tool. *BMC Bioinformatics* **14**, 128 (2013).

2. Okonechnikov, K., Golosova, O., Fursov, M., & the UGENE team. Unipro UGENE: a unified bioinformatics toolkit. *Bioinformatics* **28**, 1166–1167 (2012).

3. Edgar, R. C. MUSCLE: a multiple sequence alignment method with reduced time and space complexity. *BMC Bioinformatics* **5**, 113 (2004).

4. O’Leary, N. A. *et al.* Reference sequence (RefSeq) database at NCBI: current status, taxonomic expansion, and functional annotation. *Nucleic Acids Res.* **44**, D733–D745 (2016).
